# Overexpression of a DUF740 family gene (*LOC_Os04g59420)* imparts enhanced climate resilience through multiple stress tolerance in rice

**DOI:** 10.3389/fpls.2022.947312

**Published:** 2023-01-16

**Authors:** Karikalan Jayaraman, Amitha Mithra Sevanthi, Kalappan Venkat Raman, Gitanjali Jiwani, Amolkumar U. Solanke, Pranab Kumar Mandal, Trilochan Mohapatra

**Affiliations:** ^1^ Indian Council of Agricultural Research (ICAR) - National Institute for Plant Biotechnology, New Delhi, India; ^2^ Department of Botany, Bharathidasan University, Tiruchirappalli, Tamil Nadu, India; ^3^ Indian Council of Agricultural Research (ICAR), Krishi Bhawan, New Delhi, India

**Keywords:** rice, *LOC_Os04g59420*, DUF740 family, abiotic stress, ROS, rice blast disease

## Abstract

Functional characterization of stress-responsive genes through the analysis of transgenic plants is a standard approach to comprehend their role in climate resilience and subsequently exploit them for sustainable crop improvement. In this study, we investigated the function of *LOC_Os04g59420*, a gene of DUF740 family (*OsSRDP*-*Oryza sativa* Stress Responsive DUF740 Protein) from rice, which showed upregulation in response to abiotic stress in the available global expression data, but is yet to be functionally characterized. Transgenic plants of the rice *OsSRDP* gene, driven by a stress-inducible promoter *AtRd29A*, were developed in the background of cv. Pusa Sugandh 2 (PS2) and their transgene integration and copy number were confirmed by molecular analysis. The three independent homozygous transgenic plants (AtRd29A::OsSRDP rice transformants) showed better resilience to drought, salinity, and cold stresses, but not heat stress, as compared to the non-transformed PS2, which corresponded with their respective relative transcript abundance for *OsSRDP*. Transgenic plants maintained higher RWC, photosynthetic pigments, and proline accumulation under drought and salinity stresses. Furthermore, they exhibited less accumulation of reactive oxygen species (ROS) than PS2 under drought stress, as seen from the transcript abundance studies of the ROS genes. Under cold stress, *OsSRDP* transgenic lines illustrated minimal cell membrane injury compared to PS2. Additionally, the transgenic plants showed resistance to a virulent strain of rice blast fungus, *Magnaporthe oryzae* (*M. oryzae*). The promoter analysis of the gene in N22 and PS2 revealed the presence of multiple abiotic and biotic stress-specific motif elements supporting our observation on multiple stress tolerance. Based on bioinformatics studies, we identified four potential candidate interaction partners for *LOC_Os04g59420*, of which two genes (*LOC_Os05g09640* and *LOC_Os06g50370*) showed co-expression under biotic and drought stress along with *OsSRDP*. Altogether, our findings established that stress-inducible expression of *OsSRDP* can significantly enhance tolerance to multiple abiotic stresses and a biotic stress.

## Introduction

The abundance of genome sequence information, combined with RNA-seq and microarray data under a variety of suboptimal growth conditions, and the more prevalent stresses in the climate change scenario have unraveled many hypothetical stress-responsive genes in plants that have yet to be characterized at the molecular, biochemical, and phenotypic levels. This holds good even for the extensively researched genomic model systems like Arabidopsis (Truernit et al., 2012; Ruiz-Sola et al., 2017) and rice (Sandhu et al., 2017; Sureshkumar et al., 2019; Sevanthi et al., 2021). DUF (Domain of Unknown Function) proteins are one kind of expressed, hypothetical proteins, which are conserved across organisms, but their specific protein fragments or domains remains unknown. The number of DUF families has increased in the protein (Pfam) database due to the rapid advancement of sequencing technologies and their extensive application in plant biology. So far, approximately 4,000 DUF families (almost 22% of all families in 2019) are included in the Pfam database, and most of them are poorly characterized (Punta et al., 2012; Bateman et al., 2019; El-Gebali et al., 2019). Several plant-specific DUF proteins play an important role in various biological processes of plant growth and development, defense responses to diseases and insect pests, and adaptation responses to abiotic stresses (Palmeros-Suarez et al., 2017; Zhang et al., 2019; Kaur et al., 2020; Yang et al., 2020).

To date, a few DUF gene families have been characterized in response to abiotic stress in Arabidopsis and rice. For example, Arabidopsis *TBL3* and *TBR* genes, encoding a DUF231 domain-containing protein, are involved in secondary cell wall formation in higher plants (Bischoff et al., 2010). Arabidopsis *ESK1*, another member of the DUF231 gene family, acts as a negative regulator of cold acclimation (Xin et al., 2007). Under abscisic acid (ABA) and water-deficit stress in Arabidopsis, two RING-DUF1117 E3 ubiquitin ligase genes, *AtRDUF1* and *AtRDUF2*, were shown to be stimulated, while the suppression of their gene expression led to a reduced level of tolerance to drought stress (Kim et al., 2012). Overexpression of a wheat transcription factor *TaSRG*, which contains a DUF662 domain, in Arabidopsis and rice resulted in enhanced tolerance to salt stress (He et al., 2011). Another salt-responsive gene, *TaSRHP*, containing a DUF581 domain from wheat in transgenic Arabidopsis plants, showed enhanced tolerance to both salt and drought stresses (Hou et al., 2013). Under optimal conditions, the sorghum (*SbSGL*) gene encoding the DUF1645 protein family has been shown to be involved in the regulation of seed development and yield in rice (Zhang et al., 2018). The mutant of the DUF1517 family gene in *Arabidopsis thaliana* exhibited enhanced sensitivity to cold stress, while the heterologous expression of *AmDUF1517* in *atduf1517* Arabidopsis mutants significantly rescued their cold sensitive phenotypes (Gu and Cheng, 2014). Also, the overexpression of cold stress-responsive *AmDUF1517* has significantly enhanced tolerance to various stresses in transgenic cotton (Hao et al., 2018).

Overexpression of DUF1644 protein family genes from rice, *OsSIDP361* and *OsSIDP366*, improved tolerance to salt and drought stresses in transgenic rice (Guo et al., 2016; Li et al., 2016). While overexpression of the *OsDSR2* gene of the DUF966 family from rice has been demonstrated to act as a negative regulator of abiotic stress *via* ABA signaling (Luo et al., 2014), *OsSGL*, a grain length-enhancing gene encoding a protein with a DUF1645 domain, is shown to act as a positive regulator of drought stress tolerance in transgenic rice and Arabidopsis (Cui et al., 2016). Zhong et al. (2019) extensively analyzed the expression pattern of 12 genes in the *OsDUF668* family and revealed that all the genes were consistently upregulated under drought stress, while four of them were also upregulated in response to rice blast disease. Likewise, in another study on rice, Li et al. (2018) examined various members of the *OsDUF810* family. Among them, *OsDUF810*.*7* was found to be involved in salt and drought stress tolerance. Furthermore, the functions and expression levels of several DUF genes in rice, such as *OsDUF866*, *OsDUF946*, *OsDUF1191*, and *OsDUF617*, have been reported under various abiotic stresses and ABA treatment (Li et al., 2017a; Li et al., 2017b; Lv et al., 2019; Li et al., 2021). Apart from stress regulation, some of the DUF family proteins are involved in controlling lemma and palea development and leaf rolling in rice (Li et al., 2012; Yang et al., 2016) and the regulation of sexual reproduction and polar growth of plant cells in Arabidopsis (Jones-Rhoades et al., 2007; Cao et al., 2010). Still, several DUF domain-encoding genes remain uncharacterized in rice. Based on putative functional annotation, 133 DUFs containing protein-expressing genes in rice are listed in the MSU database. Thus, for sustainable crop productivity and maintenance of the ecological balance, it is imperative to identify and characterize genes that can impart better stress tolerance, and DUF gene family members are appropriate candidates for the same.

Here, we report, for the first time, the functional analysis of *LOC_Os04g59420* gene, a member of the DUF740 family (*OsSRDP-Oryza sativa* Stress-Responsive DUF740 Protein) in rice. As the *OsSRDP* gene (Pfam: PF05340) was found to be upregulated under one or more abiotic stresses in the publicly available genome-wide expression data of rice (Sandhu et al., 2017), this gene was chosen for functional analysis. We have cloned this gene from a drought- and heat-tolerant rice cultivar, Nagina22 (N22), under the transcriptional control of stress-inducible promoter *AtRd29A* and developed transgenic plants in the background of a drought-susceptible rice cultivar Pusa Sugandh 2 (PS2) and assayed the transgenic plants under diverse abiotic stresses and a biotic stress.

## Materials and methods

### Identification of stress-responsive gene(s) for functional validation

Two publicly available genome-wide expression datasets, E-MEXP-2401 and GSE6901, from rice genotypes N22 and IR64, pertaining to different abiotic stress treatments, were analyzed to identify differentially expressed genes using the standard RMA approach and GEO2R scripts in the limma package v3.28.2, as reported earlier (Jayaraman et al., 2021). Of the seven “expressed protein” genes upregulated in N22 and IR64 in the genome-wide and gene-specific expression analysis, *LOC_Os04g59420* showed upregulation under drought stress in both genotypes; in addition, it also showed upregulation under salinity and cold stresses in IR64 (Jain et al., 2007; Lenka et al., 2011). This gene was also found to be drought stress responsive in cv. IR20, from the meta-analysis of drought stress-specific microarray data in rice (Sandhu et al., 2017; http://14.139.229.201/RiceMetaSys). Hence, *LOC_Os04g59420* gene encoding for a DUF740 gene family was selected for functional characterization through gene complementation under stress-inducible expression for drought and other major abiotic stresses as well as a major biotic stress, rice leaf blast caused by *M. oryzae*.

### Bioinformatics analysis of *LOC_Os04g59420*

The nucleotide and protein sequence of *LOC_Os04g59420* were obtained from the MSU Rice Genome Annotation Project Database and Resource (RGAP) (http://rice.plantbiology.msu.edu). Since this gene was annotated as “expressed protein” in RGAP and lacked proper annotation in the RAP-db database (https://rapdb.dna.affrc.go.jp/), the Pfam (http://pfam.sanger.ac.uk/) and InterPro (http://www.ebi.ac.uk/Tools/pfa/iprscan/) databases were utilized to search for information about this gene. To determine the molecular weight (MW) and theoretical isoelectronic point (*pI*) of *LOC_Os04g59420*, the ProtParam tool available in the ExPASy server was used (http://web.expasy.org/protparam). Furthermore, this gene was investigated for its functional relationships using a co-expression analysis network, Ricefrend (https://ricefrend.dna.affrc.go.jp/). We also looked for transposon insertion, if any, in the target gene locus from the database, Rice Transposon Insertion Polymorphism Information (RTRIP), available in the public domain (Liu et al., 2020; http://ibi.zju.edu.cn/Rtrip/index.html). We further carried out the phylogenetic analysis of the *LOC_Os04g59420*-like genes of rice using MEGA10 software in terms of their protein domains.

### Assessment of the *OsSRDP* gene and its promoter sequences between N22 and PS2 genotypes

The reference sequence of the *OsSRDP* gene was derived from the MSU-RGAP (http://rice.plantbiology.msu.edu). Seven pairs of overlapping primers were designed covering the ~2-kb upstream promoter region and the ~1.4-kb genic region of the *OsSRDP* gene using the Primer Quest Tool software (**Supplementary Table 1**). PCR amplification was carried out using the PrimeStar GXL DNA polymerase (Takara Bio Inc, Japan) according to the manufacturer’s protocol and the obtained PCR products were purified using the NucleoSpin PCR purification kit (Macherey-Nagel gmbh & co.kg, Germany). The quantified PCR products were sequenced by Applied Biosystems 3700 XL Genetic Analyser (Applied Biosystems, USA) using standard procedure. The sequence data generated were assembled into contigs by using BioEdit Software version 7.2.6.1 (Hall, 1999). Detection of single-nucleotide polymorphism (SNP) and insertion/deletion (InDels) was carried out by comparing the *OsSRDP* sequence between N22 and PS2 as (1) they are well-known contrasts for abiotic stress tolerance (Jayaraman et al., 2021) and (2) they were the donor and WT genotypes used in the present experiment. The 2-kb upstream sequence of the *OsSRDP* gene from N22 and PS2 obtained was used as an input in the PlantCARE (Lescot et al., 2002) for cis-acting regulatory elements analysis. To identify the positions of exons and introns, amino acid sequences, and gene structure, the gene prediction software FGENESH (www.softberry.com) was used. The protein sequences were also submitted to the online server I-TASSER for protein structure prediction (http://zhanglab.ccmb.med.umich.edu/I-TASSER/).

### Construction of recombinant plasmid (pC1300::SRDP) and rice transformation

The full-length coding region of *OsSRDP* was amplified from drought-tolerant rice cv. N22 using gene-specific primers (**Supplementary Table 1**) flanked by *Kpn*I and *Sal*I restriction sites, and the amplified fragment was cloned into the pGEM-T Easy vector (Promega, USA) and sequenced for confirmation. Furthermore, the complete *OsSRDP* gene fragment was excised from the pGEM-T vector by *Kpn*I and *Sal*I double digestion and sub-cloned into the pCAMBIA1300 plant transformation vector, which has a stress-inducible promoter, *AtRd29A*; NOS terminator; and *hptII* gene as a plant selection marker (**Supplementary Figure 6A**). This plant transformation construct, pCAMBIA1300-p*AtRd29A*-*OsSRDP-NosT* (pC1300::SRDP), was used for rice genetic transformation. *Agrobacterium* strain EHA105 was transformed with the pC1300::SRDP construct developed using the standard freeze–thaw method (Hofgen and Willmitzer, 1988). A drought stress-sensitive popular aromatic rice cv. PS2 was used for the *Agrobacterium-*mediated rice transformation. The entire protocol from callus induction and selection to regeneration of putative transgenic plants was followed as mentioned by Jayaraman et al. (2021). The regenerated putative transgenic T_0_ plants were acclimatized in soil and maintained at the National Phytotron Facility (NPF, ICAR - IARI, New Delhi, India) for further generation advancement.

### Molecular characterization of putative transgenic plants

The genetically transformed plants were confirmed for their transgene integration through PCR using two pairs of appropriate primers, one pair from the selection marker, *hptII*, and the second one from SRDP29A, using *AtRd29A* promoter (Forward primer) and *OsSRDP* gene sequences (Reverse primer; **Supplementary Table 1**). Southern analysis was carried out to determine the transgene copy number. Hybridization was performed using *hptII* as a probe labeled with α [^32^P]-dCTP as described previously (Jayaraman et al., 2021). For segregation analysis, T_1_ and T_2_ progenies were screened for hygromycin resistance. This allowed us to eliminate the null plants for the transgene in the T_1_ generation. The genetic segregation analysis was done by the goodness of fit (*χ* ^2^) test, and this allowed us to raise only those T_1_ plants that showed 3:1 segregation for the transgene. In the T_2_ generation, the hygromycin selection was used to identify the homozygous T_1_ plants so that only the progenies of those homozygous plants could be maintained in T_2_ and forwarded to T_3_.

### Evaluation of AtRd29A::OsSRDP transgenic lines under various abiotic stresses and optimal growth conditions

Three independent homozygous (T_3_) AtRd29A::OsSRDP transgenic lines, along with the wild-type plants (PS2), were evaluated under various abiotic stresses. All stress experiments were carried out in three independent biological replications with each replication represented by three technical replications. Thus, the total number of samples evaluated was nine for each parameter under independent stress treatments. Drought, salinity, and cold and heat stress experiments were conducted as described in Jayaraman et al. (2021) and briefly presented here. To administer drought stress, healthy seeds of the three homozygous T_3_ transgenic lines of PS2 as well as the WT (PS2) were germinated at 28°C under dark conditions for 2 days and then transplanted at three plants per pot (12-inch-diameter earthen pots) and maintained at a transgenic screen house of ICAR-NIPB, New Delhi, India. Once the plants reached active tillering stage (represented by 9 or 10 leaf stage), drought stress was imposed by withholding water for 14 days by which time the soil moisture content (SMC) had declined to 18.5%–20% compared to the initial SMC of 57.5%–60%. After drought stress, re-watering was done for 10 days for plant recovery.

For biochemical (proline, chlorophyll, carotenoids content, and ROS scavenging) and physiological (relative water content) studies, the sampling was done by collecting the three topmost leaves from three different plants in case of each replication. The samples meant for RNA isolation and biochemical tests were immediately frozen in liquid nitrogen and stored at −80°C. For relative water content (RWC) measurements, the samples were collected in pre-weighed bags and brought to the laboratory immediately for observation of fresh weight. The samples were then placed in petri dishes filled with water for turgidity measurements. RWC, total chlorophyll, carotenoids, proline, malondialdehyde (MDA) content, and relative electrolyte leakage (REL) were measured as per Jayaraman et al. (2021). ROS scavenging potential by nitrobluetetrazolium (NBT) and diaminobenzidine (DAB) staining methods in leaf tissues were executed as described by Kaur et al. (2016). SMC was determined by a gravimetric method. All the physiological and biochemical assays were carried out on each of the three replications using three technical replicates.

For imposing salt stress, the three transgenic lines and WT were grown in basal Yoshida nutrient solution as described by Yoshida et al. (1976) under controlled growth conditions. The nutrient solution was replaced every fourth day to prevent contamination. Once the seedlings attained the fourth to fifth leaf stage, they were transferred to the Yoshida medium containing 150 mM NaCl for 7 days to impose salt stress. After this, the seedlings were transferred to Yoshida medium without NaCl and the observations were made after 6 days of recovery. All the biochemical parameters except the ROS scavenging test were done, as in the case of the drought stress experiment, by collecting the second leaf from the top of each plant at three plants per replication. For both the high- and low-temperature stress treatments, the transgenic lines and the WT were initially grown in pots containing soilrite under optimal growth conditions. Once the seedlings attained the fourth to fifth leaf growth stage, they were subjected to cold stress for 12 days at 12°C with a 16-h light/8-h dark cycle. This was followed by a recovery period of 10 days. Similarly, heat stress was also imposed on four to five leaf stage old plants by exposing them to 40°C for 3 days (Jayaraman et al., 2021). The survival rate was calculated by the number of healthy green plants in the pot/hydroponic system before stress divided by the number of green plants after imposing the stress.

Simultaneously, a parallel experimental setup was also maintained under optimal growth conditions to compare the performance of AtRd29A::OsSRDP transgenic plants and WT plants. Fifteen single plants per event were used for measuring the major agronomic traits, namely, number of productive tillers, total panicle weight, grain weight, 100-seed weight, and biomass as per Tiwari et al. (2015).

### Assessment of root morphology traits

For analysis of root system architecture under suboptimal conditions, drought stress was imposed on 21-day-old seedlings of WT and three homozygous (T_3_) AtRd29A::OsSRDP transgenic lines, by withholding water for 7 days. The images of the root samples were scanned, and extracted in tiff format, and analyzed for root morphology parameters using WinRhizo software as described by Sevanthi et al. (2021).

### Evaluation of AtRd29A::OsSRDP transgenic lines under *M. oryzae* infection

AtRd29A::OsSRDP (T_4_) transgenic plants and the WT plants were grown in a glasshouse under optimal conditions (25 ± 2°C and 16-h-day/8-h-night cycle) to evaluate their efficacy against rice blast disease caused by *M. oryzae*. Another stable transgenic rice line, AtRd29A::OsCHI2 (Chalcone isomerase2; *LOC_Os06g10210*), developed in our laboratory (Jayaraman et al., 2021) showing multiple stress tolerance was also grown and infected along with WT and AtRd29A::OsSRDP transgenic plants. The fungal inoculums of *M. oryzae* strain *Mo-ni-0025*, was cultured on potato dextrose agar (PDA) followed by Mathur’s media as per Karkute et al. (2022). After 8–10 days of reproductive growth in Mathur’s media, 5 ml of autoclaved double-distilled water was added and used for the preparation of conidial suspension (1×10^5^ conidia/ml concentration). Fresh leaves from 21-day-old rice plants were sprayed with the suspension and observed for disease symptoms by the end of 72 hpi.

### Expression analysis of *OsSRDP* and ROS scavenging genes under multiple abiotic stresses

The expression level of the *OsSRDP* and ROS scavenging genes (*OsSOD* and *OsPOD*) under different abiotic stresses were analyzed in the AtRd29A::OsSRDP transgenic lines as well as in the WT plants using quantitative real-time PCR (qRT-PCR). As mentioned earlier, second leaves from the top were used for RNA isolation from each plant sampled. Within each replication, the RNA from the three independent samples were pooled at an equimolar concentration and used for cDNA synthesis. The qRT-PCR experiment was performed in three technical replicates. The primer details are given in **Supplementary Table 1**. Relative expression was calculated for fold change using the 2^−ΔΔCt^ method (Livak and Schmittgen, 2001). *OsActin* was used as a reference gene to normalize the expression data.

### Statistical analysis

All the experiments were conducted in a randomized block design (RBD) with two treatments (control and specific stress) in three replications. Each replication was represented by three independent plants grown in three independent pots. Each parameter under independent stress experiments were analyzed individually for their analysis of variance (ANOVA) using the MSTAT-C software. Duncan’s Multiple Range Test (DMRT) was applied to identify significant variation between transgenic lines and WT plants under control and stress conditions (*p* ≤ 0.05).

## Results

*OsSRDP* (*LOC_Os04g59420*), an unannotated and uncharacterized drought-responsive gene, identified from multiple gene expression databases (Jayaraman et al., 2021), was chosen for functional analysis, for which transgenic plants of *OsSRDP* driven by a stress-inducible promoter (*AtRd29A*) in the background of a drought-susceptible rice cv. PS2 were developed and evaluated under different abiotic stresses and a biotic stress, rice blast fungus.

### *In silico* characterization of *LOC_Os04g59420*

The gene sequence of *OsSRDP* (*Oryza sativa* Stress-Responsive DUF740 Protein, *LOC_Os04g59420*) obtained from the RGAP7 (Rice Genome Annotation Project 7) database (http://rice.plantbiology.msu.edu/) showed a cDNA of 684 bp encoding a putative protein of 227 amino acids with a calculated molecular mass of 24.7 kDa and an isoelectric point (*pI*) of 10.35. Sequence analysis using the Introproscan database revealed that the query protein contains a highly conserved DUF740 domain (PF05340), which belongs to the family of Octopus-like proteins (IPR008004). Another domain, DUF995, was also present but at a suboptimal e-value of 0.086. Interestingly, this gene had no transposon insertion in any of the 3,000 rice genotypes for which whole genome sequence information as well as transposon information is available (Liu et al., 2020; http://ibi.zju.edu.cn/Rtrip/index.html).

### Structural analysis of the *OsSRDP* gene and its promoter in N22 and PS2

**Figure 1:**
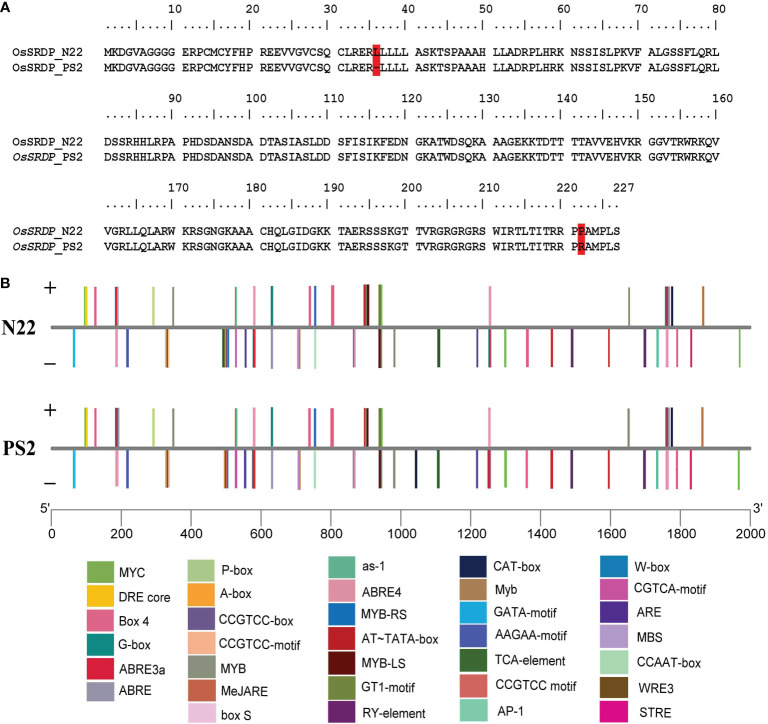
Assessment of the *OsSRDP* gene and its promoter sequences from N22 and PS2. **(A)** Variation of amino acid sequences of the *OsSRDP* gene from N22 and PS2. The red color highlight indicates the variation. **(B)** Schematic illustration of stress-related *cis*-acting regulatory elements in the promoter region of the *OsSRDP* gene from N22 and PS2. Different cis-acting elements in the 2-kb upstream region from the start codon of *OsSRDP* are illustrated by different colors in the bar chart and distributed on the sense strand and reverse strand indicated above and below the middle lines, respectively.

The gene, *OsSRDP*, and its promoter (2 kb long) were sequenced from both PS2 and N22. Sequence data revealed nine SNPs between N22 and PS2, including six transversions, one transition, and two 3-bp-long InDels in the genic sequence (**Supplementary Figure 2B**). These variations resulted in **Figure 1A**, one amino acid deletion (36: L to null) and one missense substitution (222: P to R) in PS2 as compared to N22. Thus, the alleles of the gene *OsSRDP* had distinctly different structures in N22 and PS2 (**Supplementary Figure 3**). A length difference in one of the helix, a secondary structure, was observed. Results of the ligand binding analysis showed that N22 and PS2 differed for the primary ligand pyridoxal 5’ phosphate; furthermore, an additional ligand, Zn, was predicted for N22 but not for PS2. The EC number prediction scores and subsequent GO scores, as well as the C-scores of ligand prediction, were robust for N22 but weak for PS2 (**Supplementary Figure 4**, **Supplementary Table 5**). This suggests that the alleles of *OsSRDP* in N22 and PS2 may have some functional differences. The promoter analysis of the *OsSRDP* gene revealed 11 transitions, 7 transversions, and a 2-bp-long InDels between N22 and PS2 (**Supplementary Figure 2A**). Furthermore, PlantCARE-based *cis*-acting regulatory element analysis revealed changes in the number of motifs for the salicylic acid responsiveness element (TCA-element) between N22 and PS2 (two elements in N22 against one in PS2) and the creation of one new meristem expression, related element (CAT-box) in PS2. More importantly, there were a multitude of stress-responsive elements in the promoter of *OsSRDP*; out of the 35 *cis*-regulatory elements predicted in the 2-kb-long promoter region, there were 13 abiotic stress- and 5 biotic stress-specific motifs, providing support for the stress-responsive nature of the gene (**Figure 1B**; **Supplementary Table 4**).

### Development of *OsSRDP* transgenic PS2 rice plants

Full-length coding region of the *OsSRDP* gene, 684 bp in size, was obtained by PCR amplification from cDNA of N22 (**Supplementary Figure 5**). The developed recombinant pC1300::SRDP construct was screened for gene integration by PCR using *hptII* and SRDP29A primers and confirmed with the expected product size of 1 kb and 700 bp, respectively (**Supplementary Figures 6B, C**), and restriction digestion also confirmed the integration of gene construct and gene of interest into the putative recombinant pC1300::SRDP plasmid (**Supplementary Figure 6D**). From the tissue culture regenerated plants, 22 AtRd29A::OsSRDP putative transformants (T_0_) were generated in the *indica* variety PS2 following hygromycin resistance selection (**Supplementary Figure 7**).

### Molecular confirmation of AtRd29A::OsSRDP transgenic rice plants

PCR analysis of regenerated putative T_0_ transgenic plants using SRDP29A and *hptII* gene-specific primers revealed that 13 were PCR positive (**Supplementary Figures 8A, B**). The stable inheritance of hygromycin resistance by the T_1_ progeny lines confirmed their integration into the rice genome. Only four T_0_ transgenic plants gave enough T_1_ seeds to allow genetic analysis of the transgene inheritance. Of these, three T_1_ lines, DUF-1, DUF-2, and DUF-3, exhibited a monogenic segregation ratio (3:1), while the line DUF-4 showed a digenic ratio of 15:1 (**Supplementary Table 2**). Hence, only the former three lines were forwarded to subsequent generations. The PCR analysis of the three selected single transgene integration of T_1_ and T_2_ transgenics (DUF-1, DUF-2, and DUF-3) showed the expected PCR product of approximately 700 bp and 1 kb specific to SRDP29A and *hptII* gene-specific primers, respectively (**Supplementary Figures 10, 11**), while no such amplified DNA fragments were found in the negative control (non-transgenic PS2). The T_2_ progeny of the three AtRd29A::OsSRDP transgenic lines, DUF-1, DUF-2, and DUF-3, that did not give rise to any hygromycin-susceptible plants were identified as homozygous lines (**Supplementary Figure 9**) and they were only included for Southern blot analysis, prior to phenotyping and stress tolerance assays. Southern hybridization analysis showed a single hybridization in all three events with different restriction patterns and sizes of ~4 kb, 7 kb, and 5 kb, respectively, for DUF-1, DUF-2, and DUF-3 lines, indicating independent single-gene inheritance of the transgene in each of the transgenic events. The non-transgenic plant (PS2) did not show any hybridization signal (**Supplementary Figure 8C**). These three homozygous and single-copy AtRd29A::OsSRDP transgenic lines were analyzed further in the T_3_ generation for the transcript analysis of the transgene, and the evaluation of abiotic stress-relevant physiological and biochemical traits under drought, salinity, and cold stresses. T_4_ generation transgenic plants were assayed for resistance to rice blast fungus, *M. oryzae*.

### Analysis of *OsSRDP* gene expression under various abiotic stresses

We analyzed the transcript level of the *OsSRDP* gene by quantitative RT-PCR in all the three independent T_3_ transgenic rice lines carrying stable single copy chromosomal integration of the transgene and their corresponding WT plants under abiotic stresses to understand the functional role of *OsSRDP*. Under drought stress, the transcript level of the *OsSRDP* gene in AtRd29A::OsSRDP transgenic rice lines increased by 9-fold (DUF-1), 7.5-fold (DUF-2), and 11-fold (DUF-3) as compared to WT plants (**Supplementary Figure 12A**). Similarly, under salt stress, the expression level of the *OsSRDP* was 1.3-2.3 fold higher in transgenic rice lines compared to the WT plants (**Supplementary Figure 12B**). When plants were exposed to cold stress, the *OsSRDP* expression in DUF-1, DUF-2, and DUF-3 was 5.6, 7.7, and 3.2-folds higher as compared with WT plants, respectively (**Supplementary Figure 12C**).

Since these results indicated only the expression of *OsSRDP*, for which an endogenous copy is also available in the WT as well as the transgenic plants, we compared the expression differences under control as well as stress conditions using WT expression as the baseline. This showed that there was no change between the transgenic lines and WT plants for the target gene expression, under control conditions. However, all transgenic lines showed significantly higher expression levels (2.4-6, 1.5-2.5, and 2.1-3 fold) of transgene than WT plants under drought, salt, and cold stress, respectively (**Supplementary Figure 12D**). Both of these comparisons clearly indicated that the transgene expression was elicited by the stress-inducible *AtRd29A* promoter under drought, salinity, and cold stress conditions.

### Transgenic stress-inducible *OsSRDP* confers drought tolerance in rice at active tillering stage

**Figure 2:**
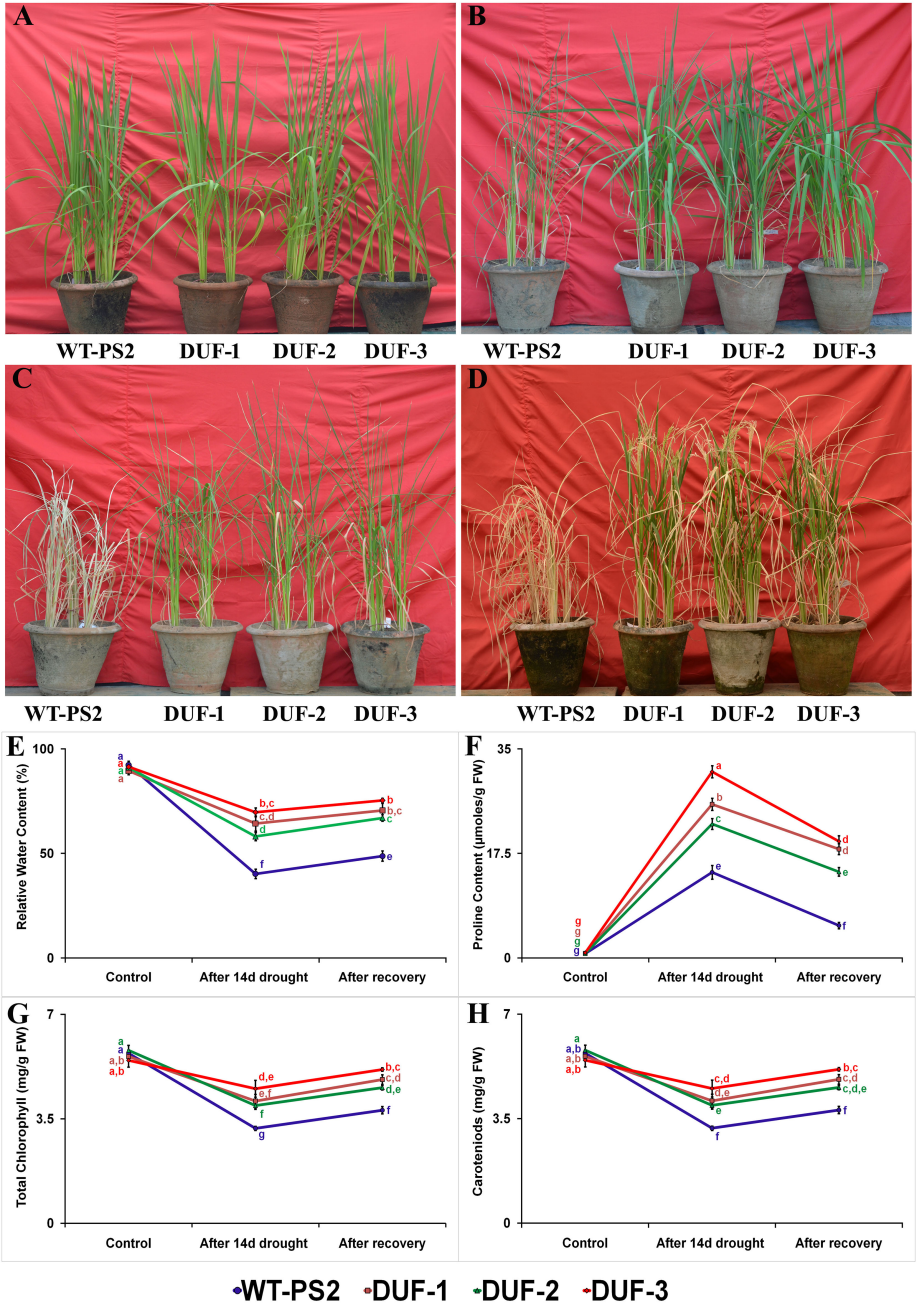
Phenotypic and physio-biochemical trait analyses of the AtRd29A::OsSRDP transgenic rice plants and WT in response to water-deficit stress. **(A)** Phenotypic appearance of WT and AtRd29A::OsSRDP transgenic rice plants at the active tillering stage under well water condition, before imposing drought stress, **(B, C)** WT and AtRd29A::OsSRDP transgenic plants subjected to drought stress for 7 and 14 days, respectively, and **(D)** recovery of plants after 10 days of re-watering. **(E)** Relative water content, **(F)** proline content, **(G)** total chlorophyll, and **(H)** carotenoids after 14 days of drought stress. Each value is the average of three independent biological replicates and the vertical bar indicates ± SD. Standard error of means (SD/√N; *N* = 3) are used as error bars, and alphabets above the vertical bars represent statistically significant differences (Duncan’s Multiple Range Test: *p* ≤ 0.05) between WT and transgenic lines.

To assess the function of stress-inducible expression of *OsSRDP* on drought tolerance in rice, drought stress was imposed on three independent T_3_ transgenic lines along with the WT plants. Under well-watered conditions, no morphological differences could be observed between the WT and transgenic AtRd29A::OsSRDP lines (**Figure 2A**). WT plants showed drought stress symptoms of leaf rolling and wilting within 7 days, while AtRd29A::OsSRDP transgenic lines remained healthy and were able to retain turgidity without any stress symptoms during this short stress period (**Figure 2B**). After 14 days of drought treatment, WT plants underwent either severe wilting or died (completely dried up), while the transgenic plants remained green, though they did show leaf rolling and wilting (**Figure 2C**). Following 10 days after re-watering, all the AtRd29A::OsSRDP transgenic plants recovered more vigorously, whereas just one or a few leaves of WT plants recovered greenness (**Figure 2D**). Both the WT and the AtRd29A::OsSRDP plants maintained RWC within the range of 89%–92.5% in optimal growth conditions, which declined to 58%–70% RWC in the transgenic plants and 40% in the WT plants after 14 days of drought stress (**Figure 2E**). The AtRd29A::OsSRDP transgenic lines DUF-1 and DUF-3 showed a higher RWC than did the DUF-2 lines before and after water stress. Ten days after re-watering, RWC increased up to 67%–75% in all the transgenic plants as compared to WT plants (49%), whose leaves had almost dried out. Similarly, degradation of photosynthetic pigments in AtRd29A::OsSRDP transgenic plants ranged from 17% to 34%, while it was 45% in WT plants (**Figures 2G, H**). After 10 days of re-watering, AtRd29A::OsSRDP transgenic plants exhibited a higher quantum of photosynthetic pigments (8%–27%) compared to WT plants (10%). AtRd29A::OsSRDP transgenic rice plants showed 18, 14, and 20-folds more accumulation of proline in the DUF-1, DUF-2, and DUF-3 lines, respectively, after 14 days of water-deficit stress (**Figure 2F**). They also showed a lesser reduction of proline content (1.4-1.6 fold) than WT plants (2.6 fold), after 10 days of re-watering.

### Analysis of root system architecture (RSA) in AtRd29A::OsSRDP transgenic plants under drought stress

**Figure 3:**
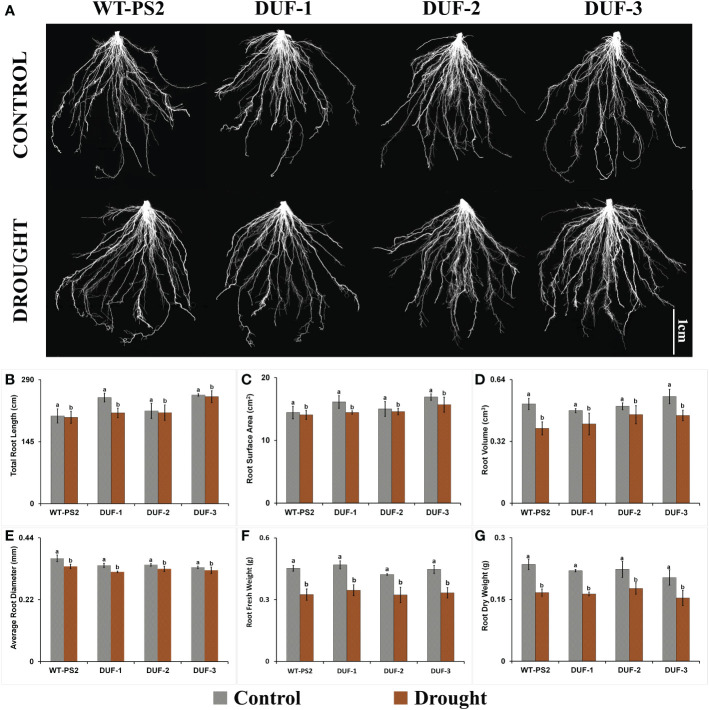
Root system architecture of AtRd29A::OsSRDP transgenic lines and WT plants under drought stress. **(A)** Comparative root architecture system images of WT and three AtRd29A::OsSRDP transgenic lines (DUF-1, DUF-2, and DUF-3) under control and drought stress conditions. All root images were captured using a flatbed scanner (Epson Perfection V700) with a resolution of 400 dpi on 28-day-old seedlings. **(B–G)** Comparison of root phenotyping traits between AtRd29A::OsSRDP transgenic rice lines and WT plants under control and drought stress. **(B)** Total root length, **(C)** root surface area, **(D)** root volume, **(E)** root diameter, **(F)** root fresh weight, and **(G)** root dry weight. Each value is the average of three independent biological replicates and the vertical bar indicates ± SD. Standard error of means (SD/√N; *N* = 3) are used as error bars, and alphabets above the vertical bars represent statistically significant differences (Duncan’s Multiple Range Test: *p* ≤ 0.05) between transgenic rice lines (AtRd29A::OsSRDP) and WT (PS2).

Since DUF740 has been implicated in plant development, especially on root system in Arabidopsis (Truernit et al., 2012; Ruiz-Sola et al., 2017), RSA was studied in the AtRd29A:: OsSRDP transgenic lines and WT plants under well-watered conditions as well as in response to drought stress. Interestingly, no noticeable differences could be observed between WT and transgenic plants in the root phenotype or RSA parameters, namely, total root length, diameter, surface area, and volume of root under either well-watered or moisture-deficit conditions (**Figure 3**; **Supplementary Figure 13**). As shown in **Figures 3D, E**, the root diameter and volume of root of the AtRd29A::OsSRDP transgenic lines showed a reduction of 3%–7% and 9%–18% under drought stress, respectively, which were statistically equivalent to those of WT plants (8% and 25%). Similar trends were observed in other root phenotyping traits such as total root length and root surface area in the AtRd29A::OsSRDP transgenic lines with respect to WT plants (PS2) after 7 days of water-deficit stress (**Figures 3B, C**). Fresh and dry weight of root of the AtRd29A::OsSRDP transgenic plants also showed a similar trend of statistically equivalent reduction (23.6%–26.3% and 20.9%–25.8%) akin to WT plants (28.3% and 29.1%) at the end of drought stress treatment (**Figures 3F, G**). These results showed that stress-induced expression of *OsSRDP* does not have any significant impact on enhancing the root system architecture in transgenic rice plants, even under drought stress.

### AtRd29A::OsSRDP transgenic plants showed less ROS accumulation in response to drought stress

**Figure 4:**
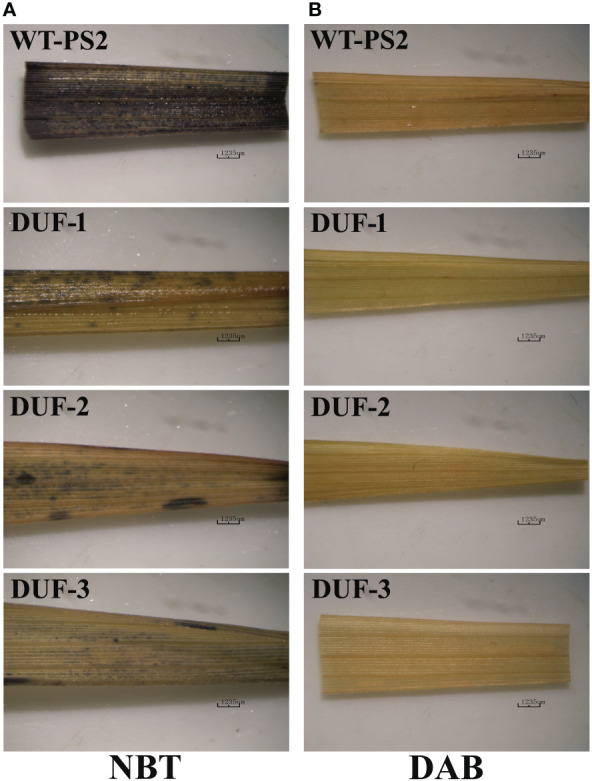
Histochemical detection of ROS accumulation in leaves of WT and AtRd29A::OsSRDP transgenic rice plants under drought stress. Detection of superoxide anion **(A)** and hydrogen peroxide **(B)** by NBT and DAB staining, respectively. Dark blue spots represent the presence of ${\text{O}}_{2}^{-}$ and brown color shows the presence of H_2_O_2_.

Accumulation of hydrogen peroxide (H_2_O_2_) and superoxide anion ( ${\text{O}}_{2}^{-}$ ) radicals in leaf tissues studied through NBT and DAB histochemical staining in the WT and the AtRd29A::OsSRDP transgenic rice lines following 2 weeks of drought stress revealed much stronger dark blue NBT staining in WT than that of the three AtRd29A::OsSRDP transgenic lines (**Figure 4A**). Likewise, WT plants showed more reddish brown DAB staining compared to AtRd29A::OsSRDP transgenic lines during water stress (**Figure 4B**). These results revealed that WT plants had a significantly higher accumulation of ROS (H_2_O_2_ and ${\text{O}}_{2}^{-}$ ) as compared to the AtRd29A::OsSRDP transgenic plants under drought treatment.

### Stress- induced expression of *OsSRDP* in rice results in improved salinity and cold stress tolerance

**Figure 5:**
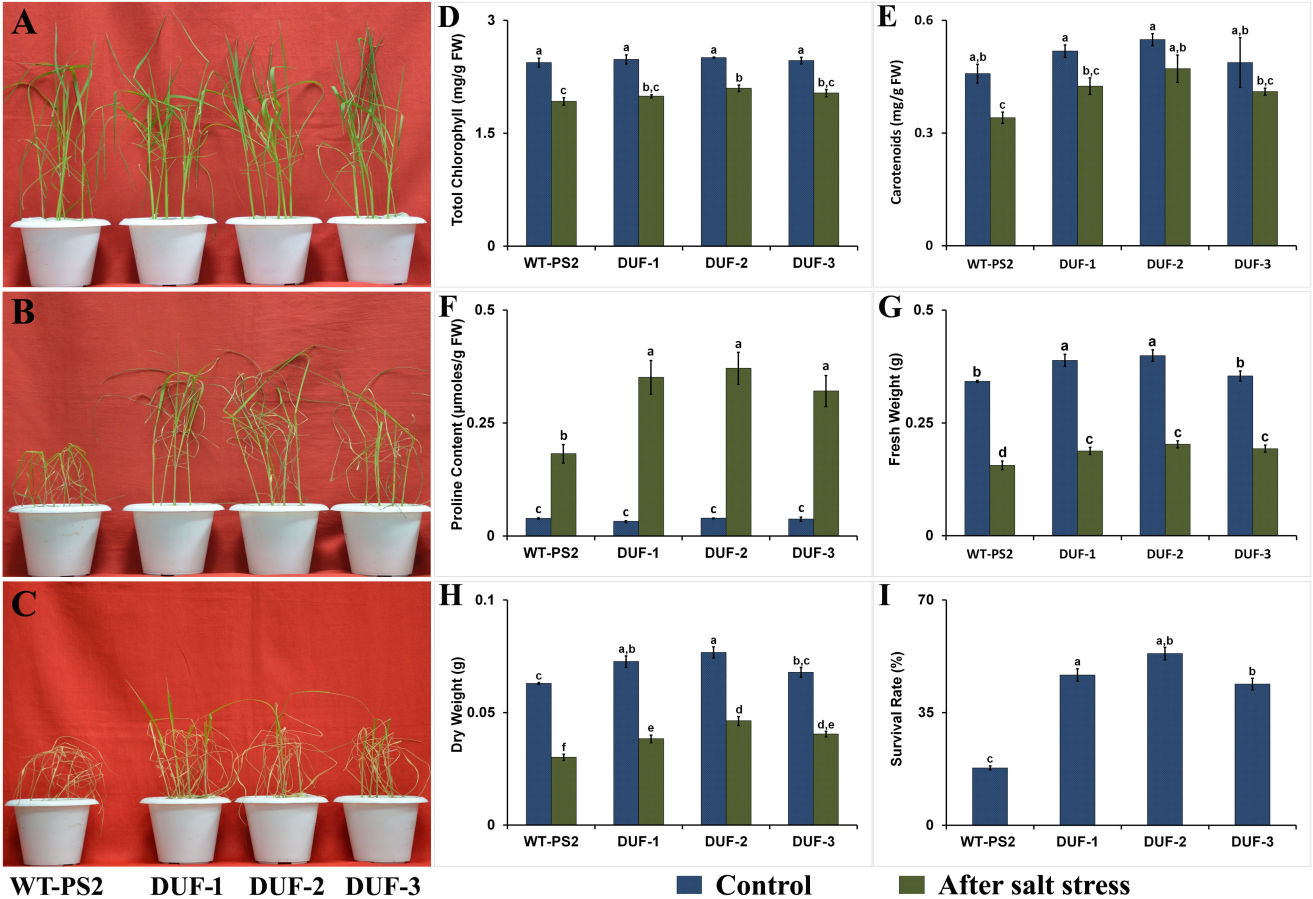
Salt stress analyses of wild-type and *OsSRDP* transgenic rice plants. **(A)** The seedlings of WT and AtRd29A::OsSRDP transgenic lines were grown in basal nutrient solution (Yoshida’s solution) under normal conditions. **(B)** Then, the fourth to fifth leaf stages of both rice seedlings were transferred into nutrient solution containing 150 mM NaCl for 7 days and **(C)** recovery for 6 days in basal nutrient solution. **(D)** Total chlorophyll, **(E)** carotenoid content, **(F)** accumulation of proline content, **(G, H)** relative fresh and dry weight of AtRd29A::OsSRDP transgenic lines and WT seedlings under control and salt stress conditions, respectively, and **(I)** the survival rate of WT and AtRd29A::OsSRDP transgenic rice lines following 7 days of salt treatment. Each value is the average of three independent biological replicates, and the vertical bar indicates ± SD. Standard error of means (SD/√N; *N* = 3) are used as error bars, and alphabets above the vertical bars represent statistically significant differences (Duncan’s Multiple Range Test: *p* ≤ 0.05) between WT and AtRd29A::OsSRDP transgenic lines.

AtRd29A::OsSRDP transgenic lines as well as WT grew well in normal YS medium and produced new leaves, which was similar in physiological appearance (**Figure 5A**). Furthermore, there were no differences in the photosynthetic pigments (total chlorophyll and carotenoids), proline content, and fresh weight among WT and AtRd29A::OsSRDP transgenic seedlings under normal YS medium. After the imposition of salt stress with 150 mM NaCl for 7 days, most of the WT plant’s leaves were severely withered, while AtRd29A::OsSRDP transgenic seedlings survived moderately without serious rolling and wilting of leaves (**Figure 5B**). More than half of the transgenic seedlings could recover by the sixth day while almost 85% of WT seedlings became pallid and died (**Figure 5C**), with survival rates of 43%–53% in the former compared to the latter (17%; **Figure 5I**). The AtRd29A::OsSRDP transgenic lines maintained less decay (8%–9%) of photosynthetic pigments than WT plants in the presence of 150 mM NaCl stress, which was not statistically significant (**Figures 5D, E**). Moreover, the transgenic seedlings showed significantly less reduction of fresh weight (45.5%–51.7%) and dry weight (40%–47.4%) as compared to the corresponding WT (54.4 and 52.3%) under salt stress (**Figures 5G, H**). Transgenic plants also showed significantly (2.3-fold) higher levels of proline accumulation compared to WT plants under salt stress (**Figure 5F**).

**Figure 6:**
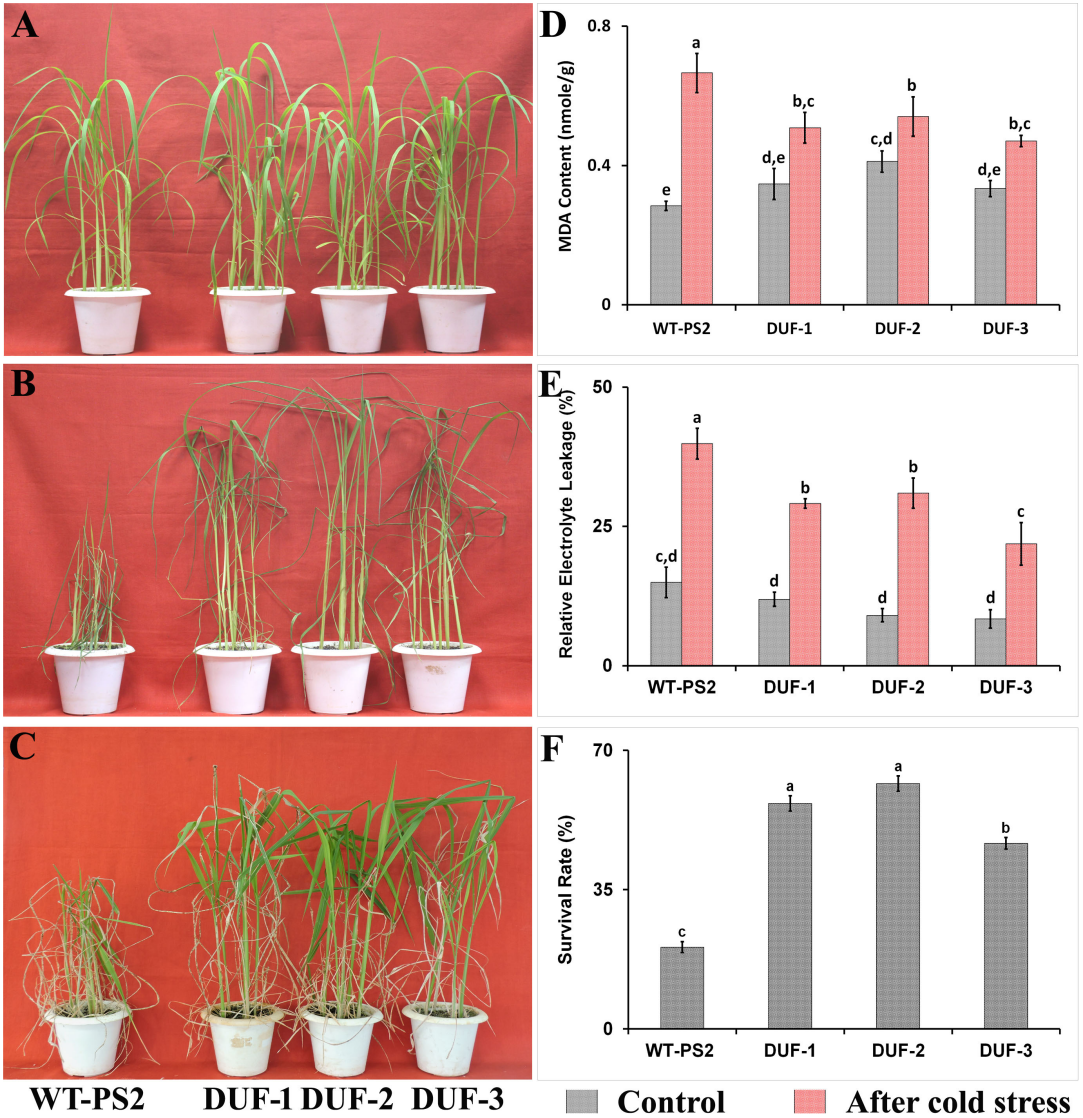
Cold stress tolerance assays of AtRd29A::OsSRDP transgenic lines with WT plants. **(A)** Phenotype of the fourth to fifth leaf stages of WT and *OsSRDP* transgenic rice seedlings, before cold treatment. **(B)** The fourth to fifth leaf stages of WT and AtRd29A::OsSRDP transgenic seedlings were under cold stress at 12°C for 12 days, and **(C)** recovery for 10 days under normal growth conditions. MDA content **(D)**, relative electrolyte leakage **(E)**, the survival rates of wild-type and AtRd29A::OsSRDP transgenic rice plants after 10 days of recovery **(F)**. Each value is the average of three independent biological replicates and the vertical bar indicates ± SD. Standard error of means (SD/√N; *N* = 3) are used as error bars, and alphabets above the vertical bars represent statistically significant differences (Duncan’s Multiple Range Test: *p* ≤ 0.05) between wild-type and AtRd29A::OsSRDP transgenic lines.

In case of cold stress tolerance, there were no variations observed in the physiological indices between the transgenic rice lines and WT plants under normal growth conditions (**Figure 6A**). After 12 days of cold stress, WT plants showed severe yellowish and wrinkled leaves, unlike transgenic lines (**Figure 6B**). In contrast, the transgenic seedlings showed moderate wilting, retaining their greenness, and showing new younger leaves upon recovery (**Figure 6C**), with an average survival rate of 47%–62%, significantly higher than that of the WT plants (21%) (**Figure 6F**). In addition, under normal conditions, we observed a similar basal level of electrolyte leakage and MDA content in the transgenic lines and WT plants. However, after 12 days of cold stress, we found >40% electrolyte leakage in WT plants, while it was<30% in the transgenic lines (**Figure 6E**). Likewise, the MDA contents of three different AtRd29A::OsSRDP transgenic lines were significantly lesser (0.6-1 fold) when compared with that of WT plants (**Figure 6D**).

All the AtRd29A::OsSRDP transgenic plants were sensitive to heat stress and wilted within 2–2½ days of exposure to higher temperature, akin to the WT plants; before heat stress, both of them appeared similar and healthy (**Supplementary Figure 14A**). As transgenic plants were highly sensitive to heat stress (**Supplementary Figure 14B**), we abandoned the experiment.

### Upregulation of ROS scavenging genes in the AtRd29A::OsSRDP transgenic lines under multiple abiotic stresses

**Figure 7:**
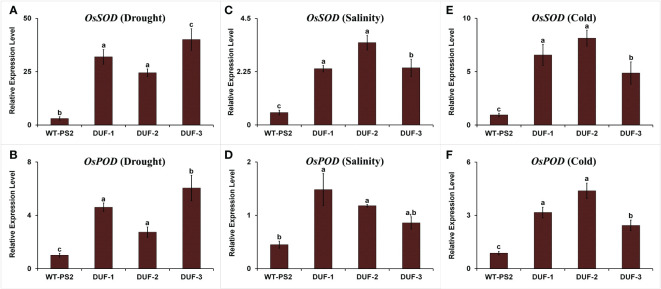
Expression level of ROS scavenging genes in WT and AtRd29A::OsSRDP transgenic lines under different abiotic stresses. Quantitative RT-PCR analyses of ROS scavenging genes *OsSOD* and *OsPOD* under drought stress **(A, B)**, salt stress **(C, D)**, and cold stress **(E, F)**. Each value represents the mean of relative expression over three biological and three technical replicates, normalized with respect to *OsActin* as an internal control. Standard error of means (SD/√N; *N* = 9) are used as error bars, and alphabets above the vertical bars represent statistically significant differences (Duncan’s Multiple Range Test: *p* ≤ 0.05) between transgenic rice lines (AtRd29A::OsSRDP) and WT (PS2).

The expression level of *OsSOD* (superoxide dismutase) and *OsPOD* (peroxidase) was significantly higher in the transgenic plants, 8-13 and 2.7-6 folds, respectively, as compared to WT plants under water-deficit stress (**Figures 7A, B**). Similarly, the expression level of the *OsSOD* gene increased more than 4.6-6.7 and 5.2-8.6 folds in AtRd29A::OsSRDP transgenic lines in comparison to the WT plants under salt and cold stresses, respectively (**Figures 7C, E**). The transcript level of the *OsPOD* gene was significantly higher by 1.9-3.3 and 2.8-5 folds under salt and cold stresses, in the transgenic rice lines (**Figures 7D, F**). Thus, the upregulation of ROS scavenging genes was found to be associated with the tolerance of AtRd29A::OsSRDP transgenic plants under multiple abiotic stresses.

### AtRd29A::OsSRDP transgenic plants showed resistance to rice blast fungus *M. oryzae*

**Figure 8:**
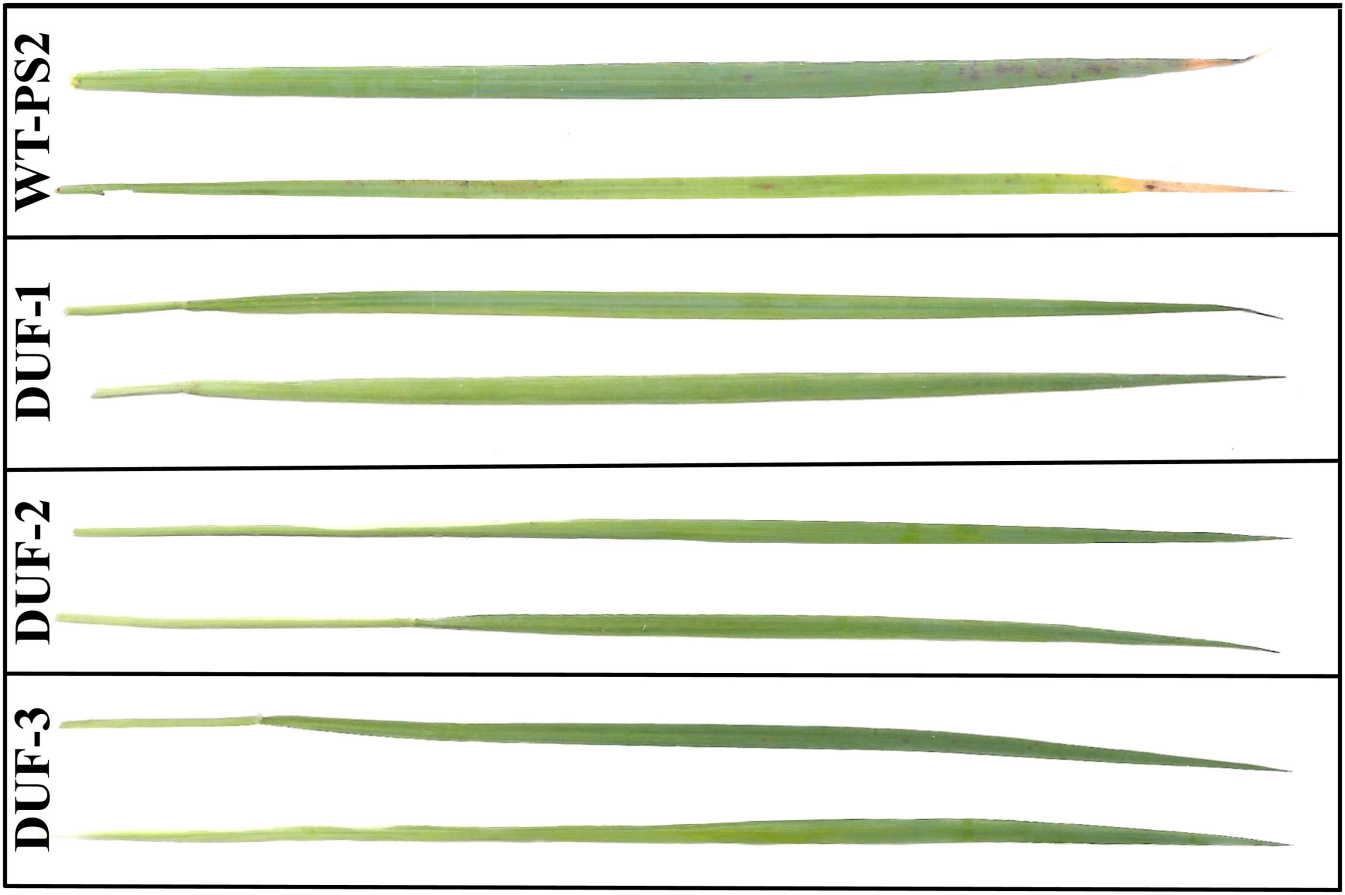
AtRD29A::OsSRDP transgenic PS2 rice plants exhibited resistance to rice blast fungus *M. oryzae*. Rice blast disease symptoms of WT (PS2) and AtRd29A::OsSRDP transgenic lines (DUF-1, DUF-2, and DUF-3) infected with the *M. oryzae* strain (Mn-ni-25) after 72 hpi. Conidial suspensions (1×10^5^ conidia/ml in 0.02% Tween-20) were sprayed onto the leaf surfaces of 21-day-old rice seedlings.

Effect of rice blast disease was evaluated on AtRd29A::OsSRDP transgenic plants along with the WT (PS2) and multiple stress tolerance AtRd29A::OsCHI2 transgenic plants (Jayaraman et al., 2021) by spraying fungal spores in the form of suspension. The disease symptoms were recorded in the form of chlorotic lesions after 72 hpi. In the case of AtRd29A:: OsSRDP transgenic plants, no lesions were observed on the leaves (**Figure 8**), whereas WT and AtRd29A::OsCHI2 transgenic plants showed lesions of size ranging from 1 mm to 4 mm diameter. The numbers of average lesions in the WT and AtRd29A::OsCHI2 plants were 7 and 10 per leaf, respectively (**Supplementary Figure 15**). These results clearly indicated that AtRd29A::OsSRDP transgenic plants were resistant to rice blast disease, while the WT (PS2) and *OsCHI2* transgenic plants were susceptible.

## Discussion

Functional genomics holds the key to precision breeding through reverse genetics tools, which has so far remained a comparatively less exploited approach in the identification of genes for the breeding of improved varieties, clones, and hybrids. Assigning function to the expressed uncharacterized genes, identified from genome and transcriptome sequencing studies, can be a worthwhile exercise in the identification of novel genes of functional value. Genes harboring domains of unknown functions (DUFs) are excellent candidates in this regard. The DUF gene family has a multitude of members within as well as across species, thus assuming evolutionary and biological significance (Finn et al., 2016; Bateman et al., 2019; El-Gebali et al., 2019). In the present study, we selected one such gene, *OsSRDP* (*LOC_Os04g59420*), with a DUF740 domain from rice, implicated in abiotic stress response, for functional validation. For transformation, we selected the allele from a drought-tolerant N22 (and source cultivar in which differential expression has been observed) with the hypothesis that it can complement the drought stress sensitivity of PS2 (a stress-sensitive variety) without compromising on growth and yield when placed under the control of a stress-inducible promoter *AtRd29A*.

The ortholog of *OsSRDP* in Arabidopsis (*At3g46990*), with 60% amino acid sequence homology to rice, has evidence for protein level expression and has been reported to be transcribed during seed germination, root, and silique development (https://www.arabidopsis.org/servlets/TairObject?accession=locus:2075606), whereas *OsSRDP* has been reported to have transcriptional evidence in germinating seeds and panicles of rice (http://rice.uga.edu/). However, there is no information on the expression of *OsSRDP* in root tissues of rice. Hence, to understand the expression pattern of this gene in various rice tissues, including roots, we estimated its transcript abundance in plumule, radicle, young root, shoot, and pre- and post**-**emergence panicles in the WT and the transgenic plants under optimal growth conditions. The results revealed that this gene is expressed in all the tested tissues both in the WT and transgenic plants, with more abundance in the transgenic plants in radicle, plumule, and root tissues; equal expression in shoot tissues; and more expression in the panicles of the WT plants (**Supplementary Figure 16**). Thus, our analyses could provide evidence for its expression in root tissues. Overall, the candidate gene’s expression in all the tissues was similar to its Arabidopsis orthologs. However, to date, this gene remains uncharacterized, though different members of the DUF740 family have been functionally elucidated (Truenit et al., 2012; Ruiz-Sola et al., 2017). We further observed that under optimal growth conditions, the transgenic plants were similar to the WT plants for all major agronomic traits, namely, number of productive tillers, total panicle weight, grain weight, 100-seed weight, and biomass (**Supplementary Figure 17**).

By Blast-P search, we identified all the DUF740 domain-encoding genes in rice, which accounted for 11 genes, of which 8 had a single DUF740 domain and the remaining 3 had two DUF740 domains (**Supplementary Table 3**). The 11 genes could be classified into three distinct clades with two, four, and five members in each of these clades (**Supplementary Figure 1**). More interestingly, except for two DUF740 genes, namely, *LOC_Os06g11510* and *LOC_Os03g08970* in clade III, none of them, including *OsSRDP* in clade IA, had any transposon element (TE) insertion, adding support to their functional relevance. The TE insertions were MITES in the upstream region (200 bp) of *LOC_Os03g08970* and Copia in the CDS of *LOC_Os06g11510*.

Since we originally found *OsSRDP* from drought stress-specific microarray data, we explored whether any of these DUF740 domain genes are implicated in drought stress response, using RiceMetaSys database (Sandhu et al., 2017). Besides *OsSRDP*, two other genes, namely, *LOC_Os03g08970* and *LOC_Os02g46420*, showed differential response under drought stress. However, the direction of response was different in these two genes; *LOC_Os03g08970* was upregulated under drought stress while *LOC_Os02g46420* was downregulated (**Supplementary Table 3**). An earlier study confirmed the expression of *OsSRDP* under drought stress in a pair of drought-sensitive (IR64) and -tolerant (N22) genotypes (Jayaraman et al., 2021). The presence of multiple stress-responsive *cis*-acting regulatory elements in *OsSRDP* also provided evidence for its stress-responsive nature (**Figure 1**; **Supplementary Figures 2, 3**). These background analyses provided us with compelling reasons for functional characterization of *OsSRDP*.

All the physiological and biochemical assays for abiotic and biotic stress tolerance studies were conducted in three independent, single-copy, and homozygous transgenic plants (T_3_ and T_4_), which ensured not only seed availability for various experiments and tests for multiple parameters but also robust results as segregation during gamete formation was ruled out. AtRd29A::OsSRDP transgenic plants showed enhanced drought tolerance as demonstrated from their RWC, proline content, photosynthetic pigments, ROS accumulation, expression of ROS scavenging enzymes, and recovery after drought stress (**Figures 2**, **4**, **7A, B**). In most plants, average initial wilting RWC is approximately 60%–70% (https://plantstress.com/leaf-relative-water-content-rwc/) and average % reduction in RWC is 20%–40% under drought stress and only severely desiccated and drying leaves show 30%–40% reduction in RWC. In rice, the daytime RWC is reported to be approximately 84%–95% across genotypes (Bunnag and Pongthai, 2013; Dien et al., 2019) and we observed a similar trend. RWC of the transgenic plants was ~58%–70% after drought stress, indicating that they are in the initial stages of wilting; in case of WT, it was 40%, showing that the WT is experiencing severe stress (**Figure 2E**). Furthermore, in terms of % reduction in RWC after stress, the WT showed a 56% reduction, which was nearly double compared to the transgenic plants (26%–36%), reflecting the extreme desiccation in the former. It is known from previous studies that the increase in proline content is directly proportional to the degree of drought stress tolerance response; however, it is also known that it comes down soon after the withdrawal of the stress (Dien et al., 2019). In our study, while the increase in proline content was directly proportional to the degree of stress tolerance, the decrease was comparatively slower even after 10 days of recovery (**Figure 2F**).

A DUF740 domain containing OCTOPUS like (OPS-like) gene from Arabidopsis, *At2g38070*, encoding for the *OPS2* gene has been shown to function in the differentiation of root protophloem, similar to the *OPS* gene (Truernit et al., 2012; Ruiz-Sola et al., 2017). Thus, we studied the RSA under control and drought conditions but could not find any gross morphological changes (**Figure 3**; **Supplementary Figure 13**). It is important to note here that the function of the specific ortholog of *OsSRDP* in Arabidopsis, *At3g46990*, has not been characterized, and no phenotype information on the insertion mutants of this gene is available yet. Though our expression analyses did confirm its expression in rice roots, the phenotype observations made on RSA suggested no role for *OsSRDP* in root development. Thus, the mechanism of enhanced drought tolerance by *OsSRDP* is not through RSA modulation. Similarly, the inflorescence of WT and transgenic plants was similar in appearance under control as well as all stress conditions. Thus, to conclude on the developmental role of *OsSRDP*, if any, knockout lines will be required. The present study rather focused mainly on the abiotic stress tolerance role of the *OsSRDP*. Among the four different abiotic stresses imposed, the AtRd29A::OsSRDP transgenic lines were susceptible only to heat stress (**Supplementary Figure 14B**), as found in our earlier study, wherein *OsCHI2* gene driven by *AtRd29A* expression also failed to show heat stress tolerance (Jayaraman et al., 2021). It could be due to the passive response of the *AtRd29A* promoter against heat stress (Yamaguchi-Shinozaki and Shinozaki, 1994; Jayaraman et al., 2021). Hence, to decipher the role of the *OsSRDP* gene in heat stress, either constitutive promoter-driven overexpression lines or heat stress specific promoter-driven transgenic lines will be required.

**Figure 9:**
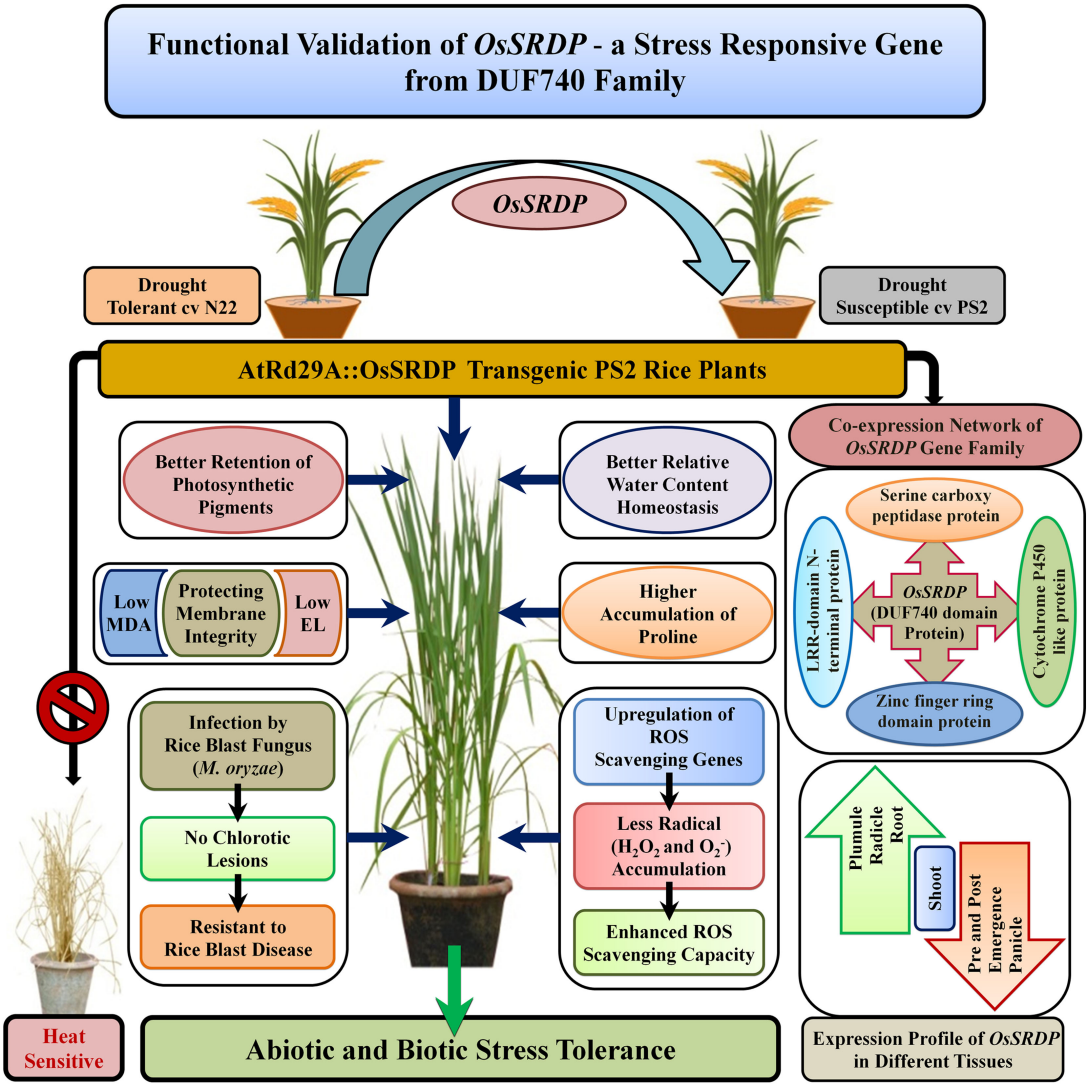
A proposed model depicting the function of the *OsSRDP* (*LOC_Os04g59420*) gene, a member of the DUF740 family, in the regulation of multiple abiotic stresses and biotic stress tolerance. Stress-inducible expression of *OsSRDP* in transgenic PS2 rice plants exhibited enhanced tolerance to drought, salt, and cold stresses through higher plant water status, photosynthetic pigments, osmo-protectant accumulation, enhanced ROS scavenging capacity through upregulation of ROS scavenging genes, lower accumulation of lipid peroxidation (MDA), and minimal cell membrane injury. In addition, the transgenic rice lines were also resistant to rice blast fungus *M. oryzae*.

A more interesting outcome from this study is that the *OsSRDP* transgenic rice plants showed enhanced tolerance to multiple stresses (**Figures 2**, **5**, **6**, **9**) including biotic stress. We have been working on characterizing unknown/uncharacterized and conserved genes for drought stress tolerance and recently reported that *OsCHI*2 from rice imparted tolerance to multiple abiotic stresses (Jayaraman et al., 2021). Hence, the *OsCHI2* transgenic rice plants developed earlier and *OsSRDP* plants developed in the present study were tested for resistance to one of the most important biotic stresses of rice, blast disease, caused by *M. oryzae*. While AtRd29A::OsCHI2 transgenic plants were susceptible to rice blast disease (**Supplementary Figure 15**), AtRd29A::OsSRDP transgenic lines showed a resistance reaction (**Figure 8**). We further checked the expression of this gene in several leaf transcriptomes of rice blast disease available in the public domain (Sureshkumar et al., 2019), but could not find any evidence for its differential expression under blast infection.

We further looked for the interacting partners of this gene using RiceFREND, which showed that *LOC_Os04g33370*, *LOC_Os05g09640*, *LOC_Os06g50370*, and *LOC_Os11g27329* were the primary interacting genes annotated as Cytochrome P450-like protein, Leucine rich repeat (LRR) domain N terminal-containing gene, Zinc finger C3HC4 RING-like domain-containing protein, and a serine carboxypeptidase protein (https://ricefrend.dna.affrc.go.jp/; **Supplementary Figure 18A**). Among them, only *LOC_Os06g50370*, a Zinc finger C3HC4 RING-like domain-containing protein, was found to be differentially expressed in the transgenic lines of Nipponbare overexpressing a resistant *Pish* gene when challenged with the Kyu77 strain of *M. oryzae* (Tanabe et al., 2014; Sureshkumar et al., 2019). Furthermore, the ortholog of *LOC_Os06g50370* from Arabidopsis is characterized as NEP-1 (Necrosis and ethylene production)-interacting protein. NEP proteins are common to three kingdoms of pathogens, leading to a typical microbe-associated molecular pattern (MAMP) to make the host susceptible to infection and colonization (Oome et al., 2014). Such NLP (NEP-1 like family) genes have been reported from *M*. *oryzae* (*Mo*NLP) and the family of *Mo*NLP has been reported to be dispensable for infection in rice, especially in susceptible host (Fang et al., 2017). The expression analysis of the *M. oryzae* challenged plants in our study showed that this was indeed the case, with the level of *LOC*_*Os06g50370* transcripts in independent transgenic rice lines being 2.5-5 folds higher than that of WT plants (**Supplementary Figure 18F**). Thus, our study indicated that a susceptible host plant transformed with *OsSRDP* could provide a sufficient effect to recognize the MAMP of the pathogen and counter its infection, most likely through its interacting partner of NEP-1-interacting protein. Though the Leucine rich repeat (LRR) domain, N terminal-containing gene, *Os05g188700*, is orthologous to an Arabidopsis ortholog, which is implicated in response to jasmonic acid response, we could not find any expression support for this gene. Thus, the consistent resistance reaction shown by all the OsSRDP transgenics could have a molecular basis in the form of *LOC_Os06g50370*.

A similar search for the expression of the interacting partners of *OsSRDP* under drought stress revealed that only *LOC_Os06g50370* was differentially expressed in a drought stress-tolerant rice cv. Dhaggadeshi (Sandhu et al., 2017). Furthermore, two other interacting partners, *LOC_Os05g09640* and *LOC_Os11g27329*, were differentially expressed under low nitrogen and drought stress in rice seedlings (Sevanthi et al., 2018). Expression analysis of primary interacting partners revealed that in the transgenic plants exposed to water-deficit stress, the expression levels of *LOC*_*Os06g50370* and *LOC*_*Os05g09640* were significantly higher in the AtRd29A::OsSRDP transgenic lines (5-8 and 12-20 folds, respectively) as compared to WT plants (**Supplementary Figures 18D, E**). No transcript-level support could be found for the rest of the two interacting partners identified (**Supplementary Figures 18B, C**). Overall, the bioinformatics and expression evidence amply supported by the biochemical data in terms of ROS scavenging capacity and accumulation osmo-protectant, proline, and better physiological parameters under different abiotic stresses thus substantiates the multiple stress tolerance nature of the *LOC_Os04g59420* gene (**Figure 9**). Development and testing of genome-edited or knockout lines will prove useful in establishing their role at the molecular and functional level.

## Conclusion

Our study has established a role for *OsSRDP* (*LOC_Os04g59420*) under multiple abiotic and biotic stress conditions. By developing and testing AtRd29A::OsSRDP transgenic rice plants, we established the drought, salinity, and cold stress tolerance ability of this gene. Its added advantage as a rice blast disease-tolerant gene makes it a novel and very useful resource in commercial and sustainable rice breeding. Based on the co-expression analysis, we have identified the plausible interacting partner in *LOC_Os04g59420*, which could be playing a key role in imparting multiple stress tolerance. Exploring the molecular mechanism of this gene using functional genomics tools can help to elucidate its function unequivocally.

## Data availability statement

The datasets presented in this study can be found in online repositories. The names of the repository/repositories and accession number(s) can be found in the article/**Supplementary Material**.

## Author contributions

KJ performed all of the experiments and data interpretation, and drafted the original manuscript. AMS helped in molecular, physiological, and biochemical experimentation; data analysis; manuscript drafting; and editing. KVR helped in the analysis of data and Southern blot analysis. GJ and AUS performed biotic stress experiment. PKM conceptualized and designed the experiments, revised the manuscript, and supervised the entire work. TM provided the original concept and edited the manuscript. All authors contributed to the article and approved the submitted version.
