# Supplementary material for: Overexpression of a DUF740 family gene (LOC_Os04g59420) imparts enhanced climate resilience through multiple stress tolerance in rice
Source: Front Plant Sci. 2023 Jan 16;13:947312. doi: 10.3389/fpls.2022.947312 (PMC9893790; doi:10.3389/fpls.2022.947312)
Supplement: Supplementary file 1 [file DataSheet_1.doc]

**SUPPLEMENTARY DATA**


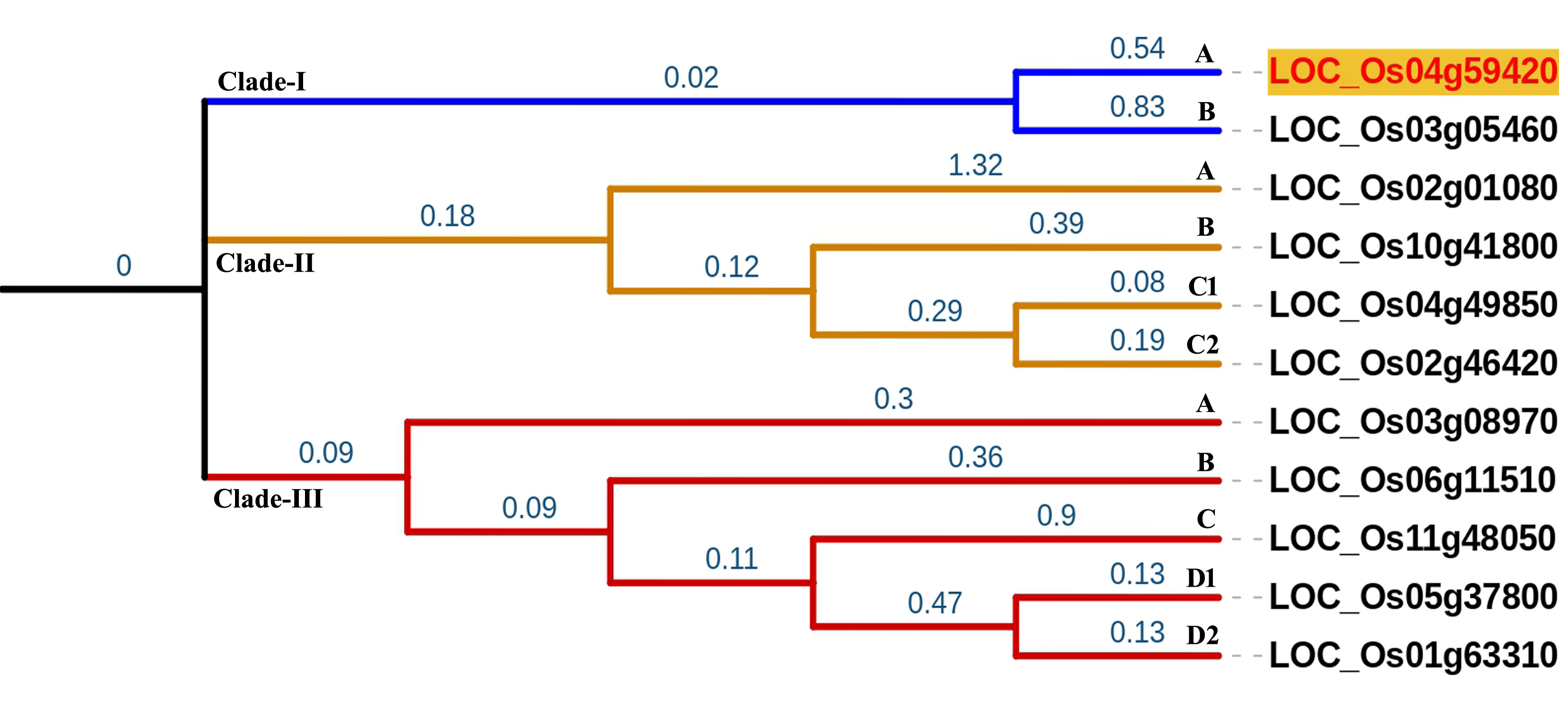


**Supplementary Figure1: Phylogenetic relationship of the eleven DUF740 gene family members present in the rice.** *LOC_Os04g59420* (*OsSRDP*) used in this study is highlighted with a red text. Phylogenetic tree was generated with amino acid sequences *via* MEGA10 software using neighbor joining method. DUF740 members are classified into three distinct clade on the basis of sequence homology as shown in different colors.


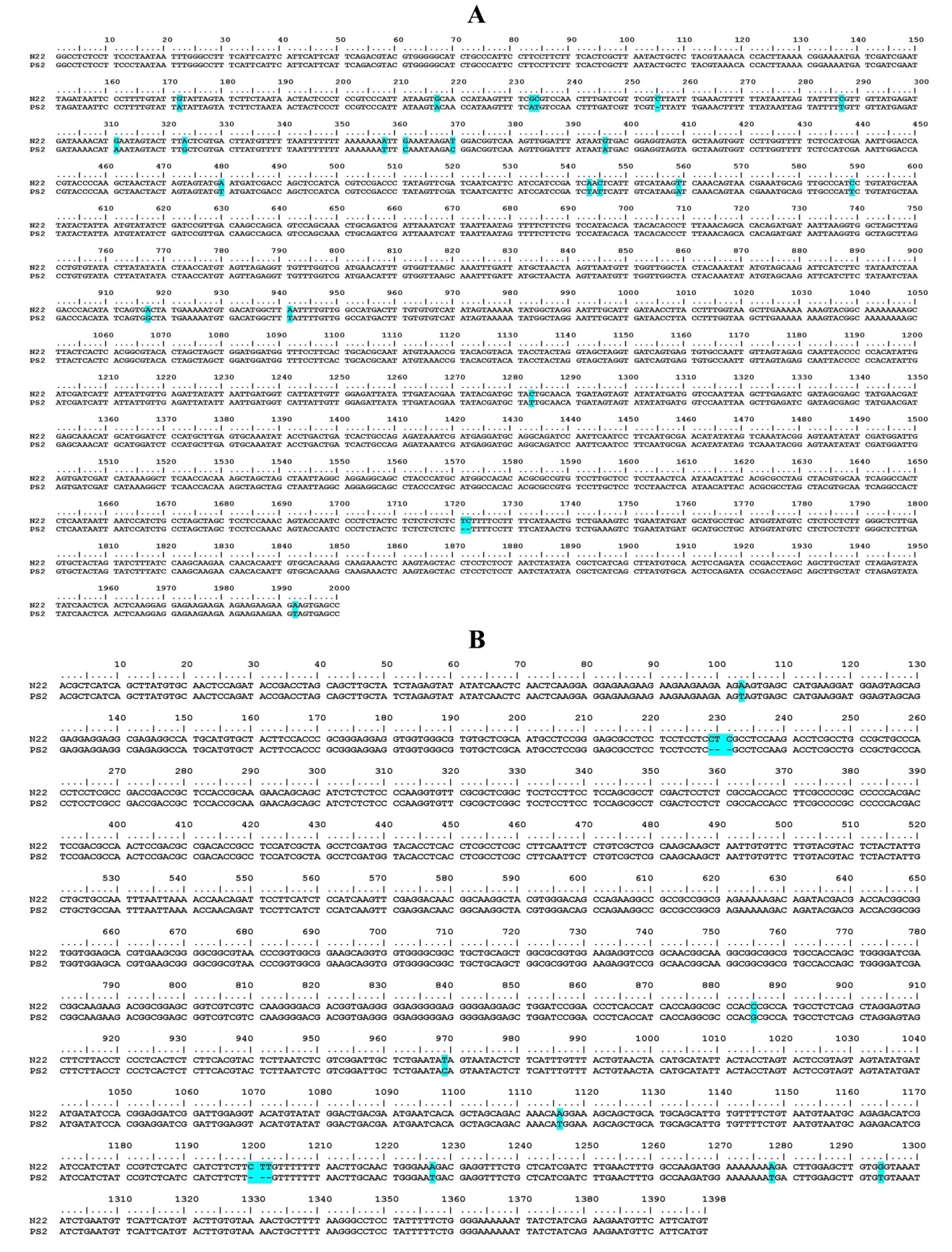


**Supplementary Figure 2: Nucleotide variation of the *OsSRDP* gene and its promoter sequences in N22 and PS2. (A)** Nucleotide sequence alignment of 2kb upstream promoter region from the start codon of the *OsSRDP* gene from N22 and PS2 genotypes, (**B**) Alignment of the *OsSRDP* gene sequences from N22 and PS2 genotypes. The Turquoise colour highlight indicates the nucleotide variation.


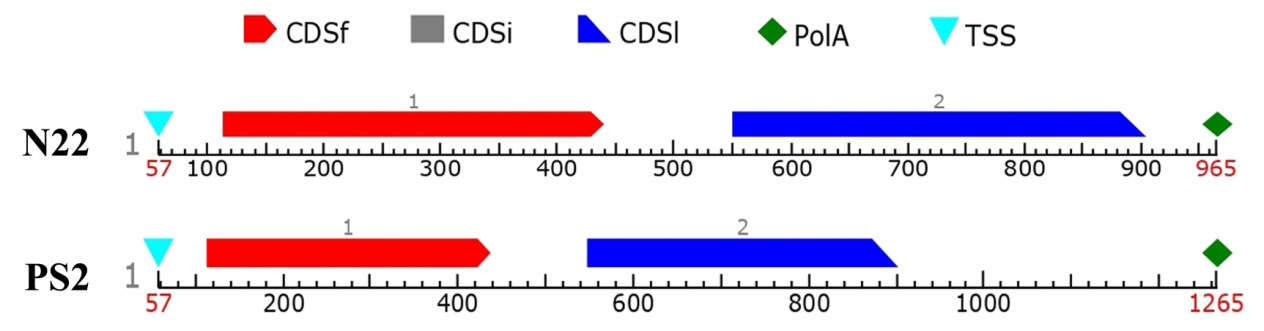


**Supplementary Figure 3:** The FGENESH prediction of *OsSRDP* gene structure from N22 and PS2. CDSf-first (starting with start codon) coding exon, CDSi-internal exon, CDSl-last coding segment, PolA-position of polyadenylation, TSS-position of transcription start (TATA-box position).


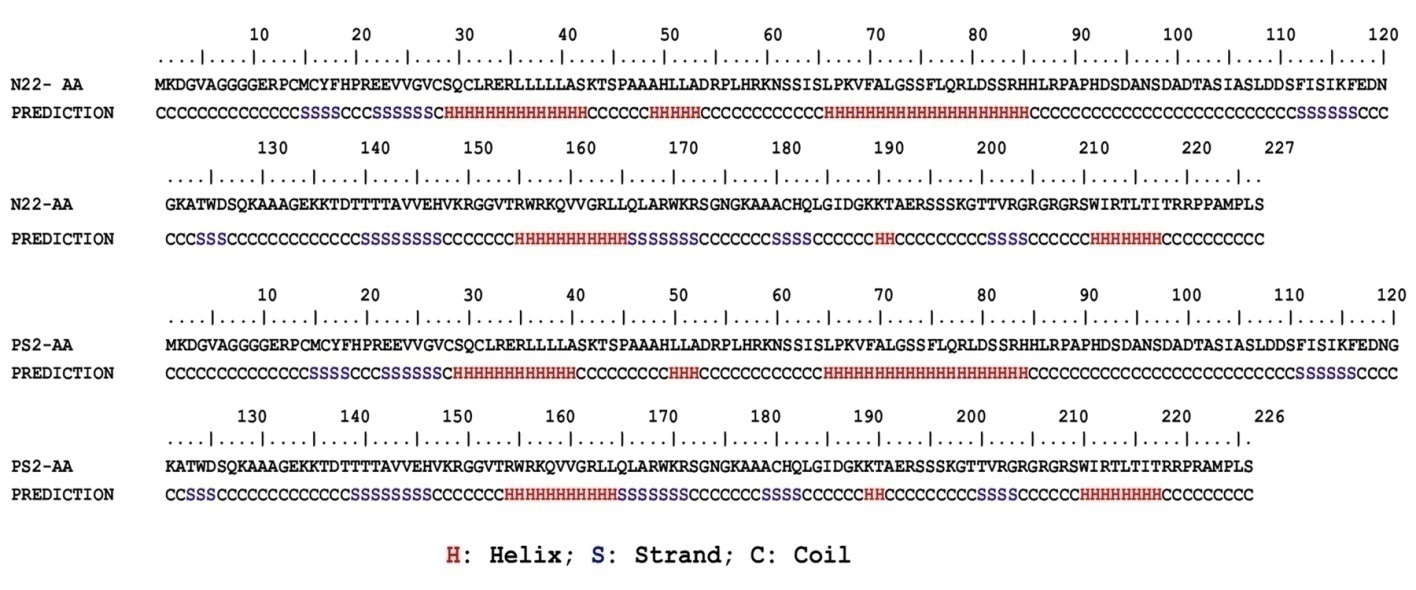


**Supplementary Figure 4: Comparisons of predicted protein structure of OsSRDP in N22 and PS2.**


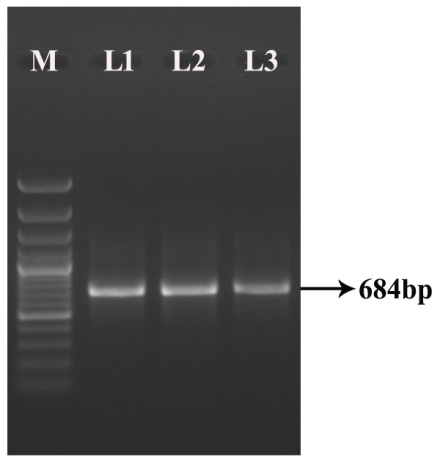


**Supplementary Figure 5: PCR analysis showing amplification of full length coding region of *LOC_Os04g59420* (*OsSRDP*) gene from rice cv. Nagina22**. Lane M **-**100bp ladder plus DNA marker, Lane 1**-**3**-**PCR amplified product of *LOC_Os04g59420*.


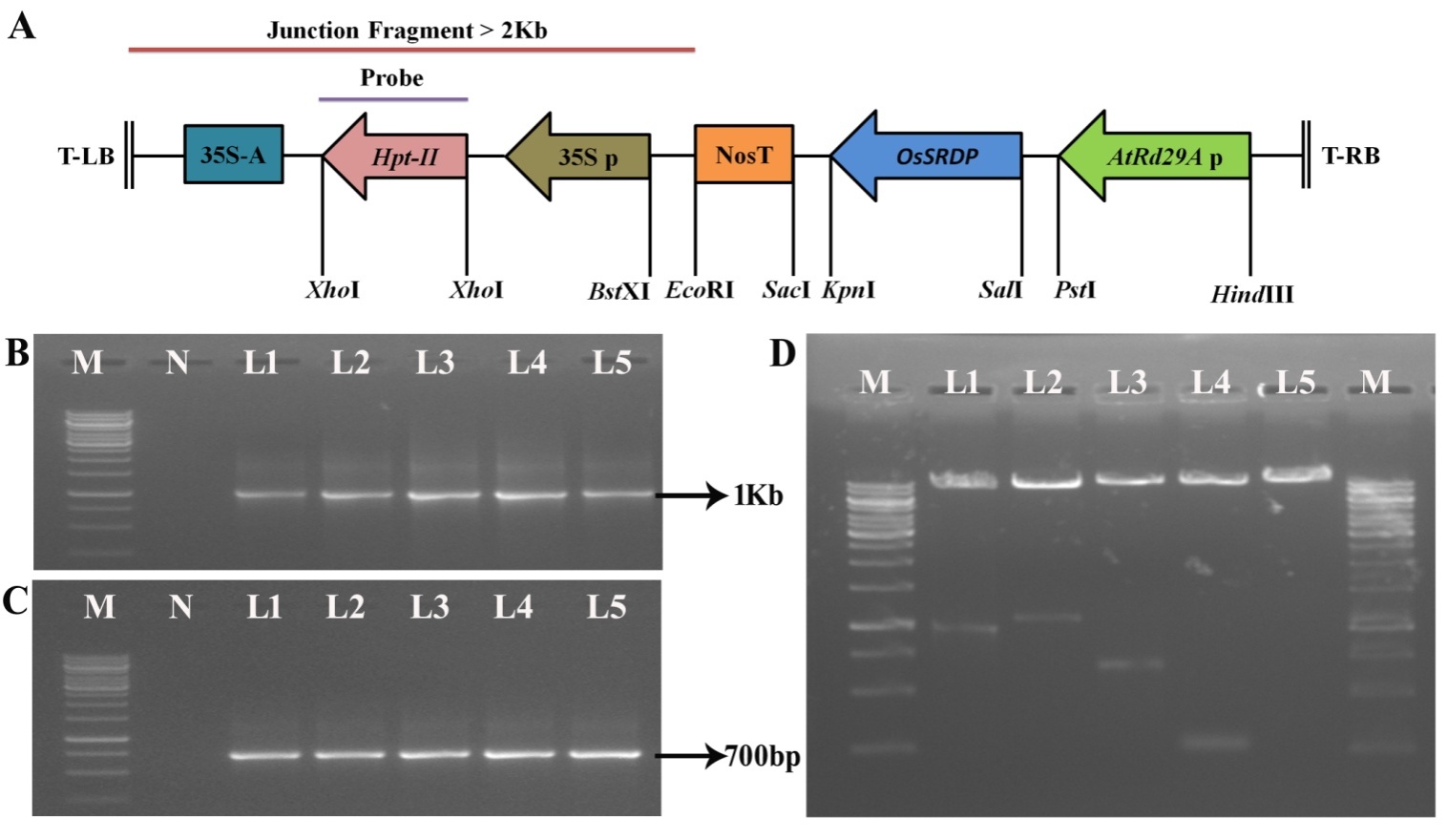


**Supplementary Figure 6: Schematic representation of the vector construct and confirmation of pC1300::SRDP construct.** (**A**) Linear map of expression cassette of the *OsSRDP* gene driven by under the control of stress inducible promoter *AtRd29A* used for genetic transformation of rice variety Pusa Sugandh 2. (**B and C**) PCR confirmation of recombinant pC1300::SRDP constructs using *hptII* and SRDP29A primers, respectively. Lane M-1kb ladder plus DNA marker, Lane N-Negative control, Lane 1-5-putative positive clones showed amplification of 1kb and 700bp products. **(D**) Restriction analysis of putative recombinants plasmid (pC1300::SRDP) for the presence of gene cassette and gene of interest. M-1kb ladder plus DNA marker, Lane 1-Digestion of putative positive clone with *Hin*dIII and *Pst*I restriction enzymes to confirm the presence of *AtRd29A* promoter (~970bp) in pC1300::SRDP vector, Lane 2-*Xho*I digestion releases hygromycin gene (~1.1kb) from putative positive clone with vector backbone (~9.9kb), Lane 3-Digestion of putative positive clone with *Kpn*I and *Sal*I restriction enzymes to confirm the presence of *OsSRDP* gene (~700bp) in pC1300::SRDP vector. Lane 4-*EcoR*I and *Sac*I digestion of putative positive clone to releases NOS-terminator (~250bp) and vector backbone (~10.6kb), Lane 5-*Hin*dIII digestion linearizes the pC1300::SRDP construct.


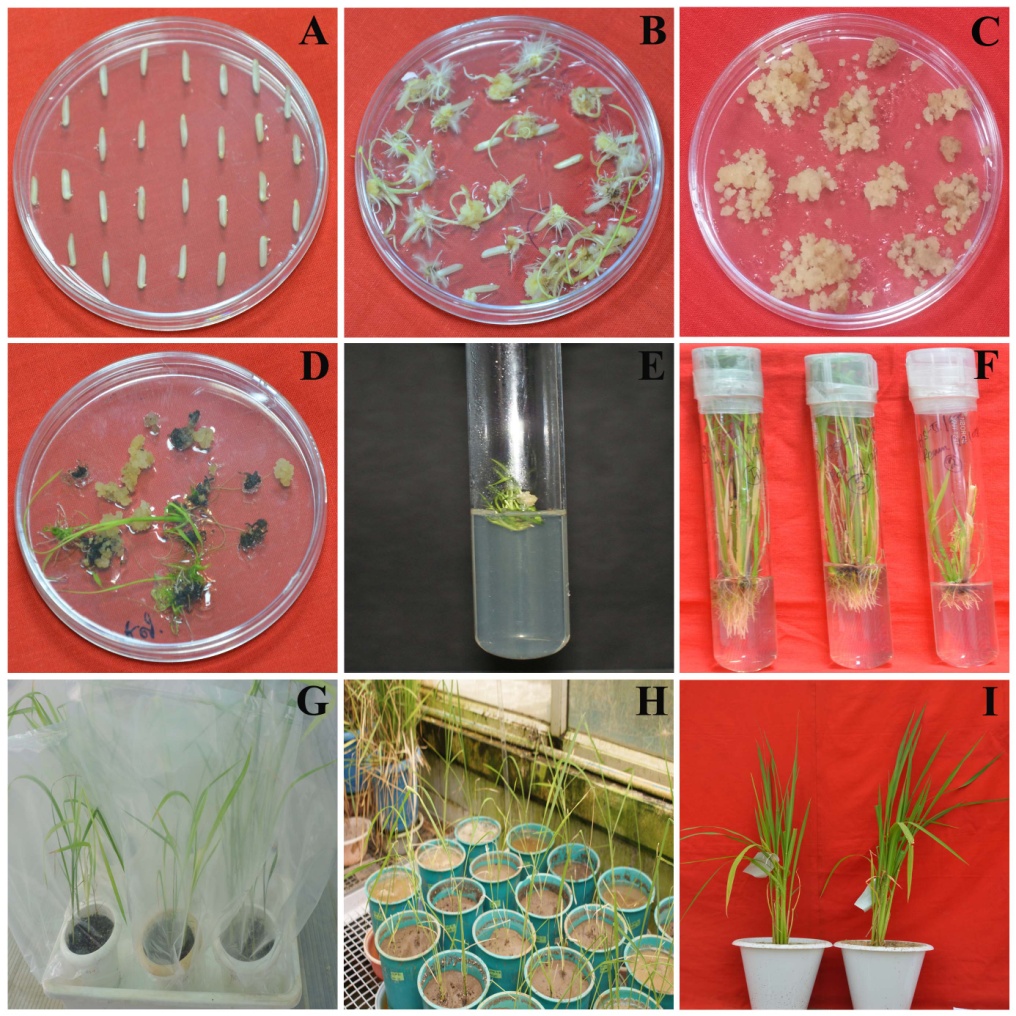


**Supplementary Figure7: Development of AtRd29A::OsSRDP transgenic Pusa Sugandh 2 plants**

(**A-B**) Seed inoculation and callus induction from mature PS2 seeds, (**C**) Proliferating hygromycin resistant calli on selection medium, (**D**) Plantlets regenerated from hygromycin resistant calli on regeneration medium, (**E**) Regeneration of green plantlets on shooting medium, (**F**) Putative transgenic in rooting medium, (**G**) Hardening of putative *OsSRDP* transgenic in soilrite, (**H**) Transplanted putative T_0_ AtRd29A::OsSRDP transgenic plants in soil at phytotron, (**I**) Matured (T_0_) transgenic plants of Pusa Sugandh 2 grown in phytotron glass house.


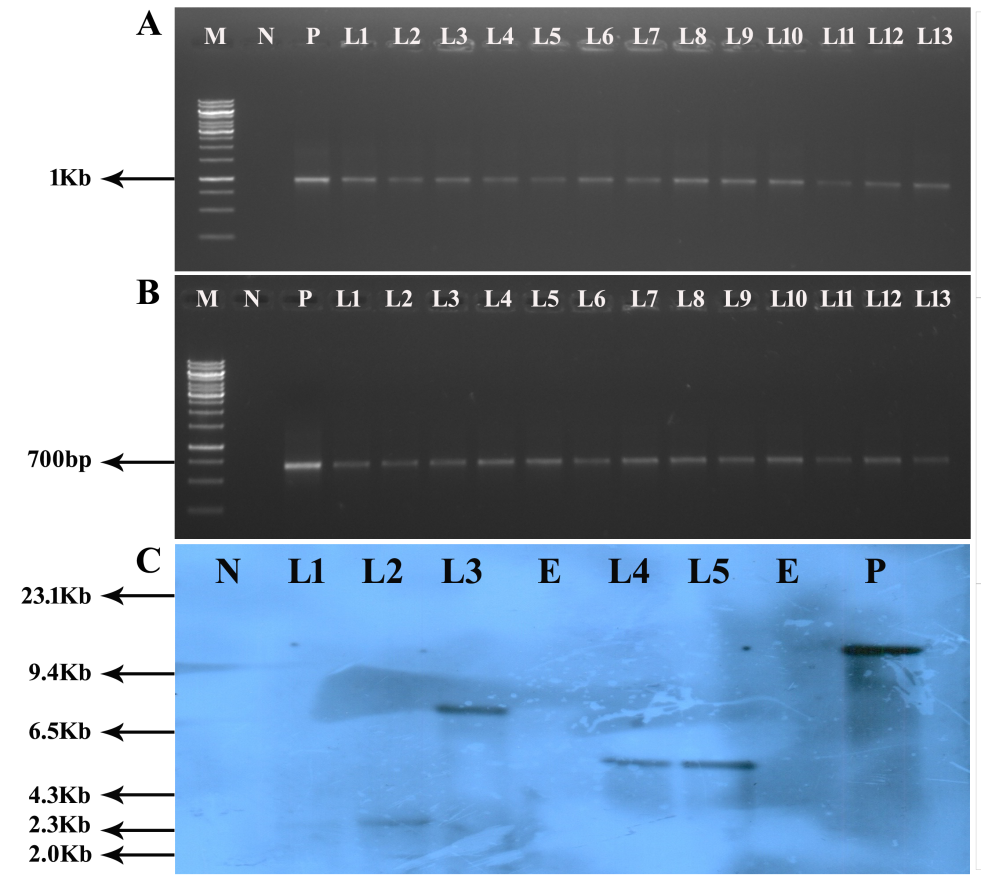


**Supplementary Figure 8: Molecular characterization of putative AtRd29A::OsSRDP transgenic lines.**

(**A**) PCR analysis of putative T_0_ transgenic AtRd29A::OsSRDP lines with *hptII* specific primers amplified a 1kb product; (**B**) PCR confirmation of putative T_0_ transgenic AtRd29A::OsSRDP transgenic lines with SRDP29A primer (*AtRd29A* promoter forward primer and reverse primer from the middle of the *OsSRDP* gene) amplified a 700bp product, Lane M-1kb ladder plus DNA marker, Lane N-Negative control (WT-PS2), Lane P-Positive control, Lane 1-13-Putative AtRd29A::OsSRDP transgenic plants, (**C**) Southern hybridization analysis of T_3_ transgenic AtRd29A::OsSRDP rice plants using *hptII* as a probe. Lane N-Negative control (Non transgenic-PS2), Lane 1-2-Putative AtRd29A::OsSRDP transgenic line DUF-1, Lane 3-Putative AtRd29A::OsSRDP transgenic line DUF-2, Lane 4-5-Putative AtRd29A::OsSRDP transgenic line DUF-3, Lane E-Empty and Lane P-Positive control (Linearized pC1300::SRDP plasmid by *EcoR*I).


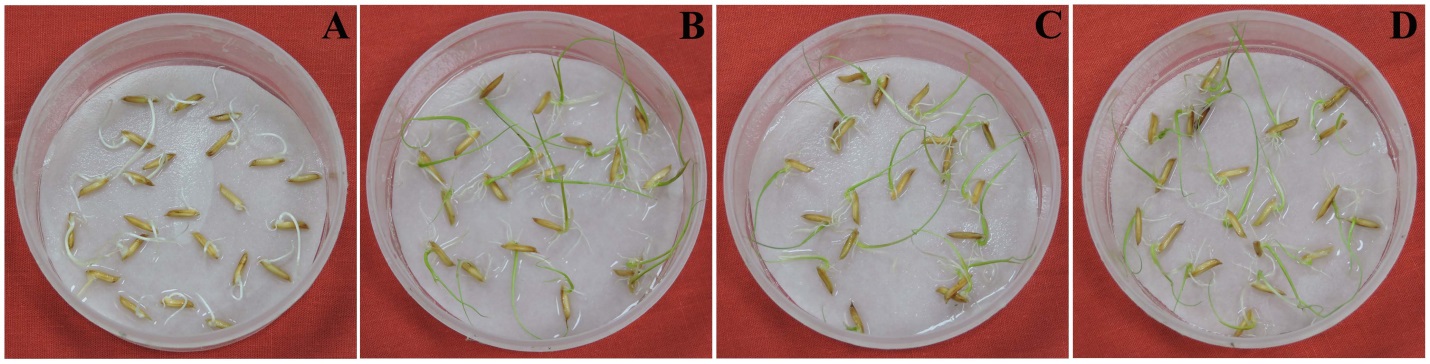


**Supplementary Figure 9: Hygromycin screening of homozygous T_2_progeny of three transgenic events.** (**A**) All of them non transgenic (PS2) seedlings were died; (**B-D**) T_2_ progeny of all AtRd29A::OsSRDP transgenic rice events were survived in DUF-1, DUF-2 and DUF-3, respectively.


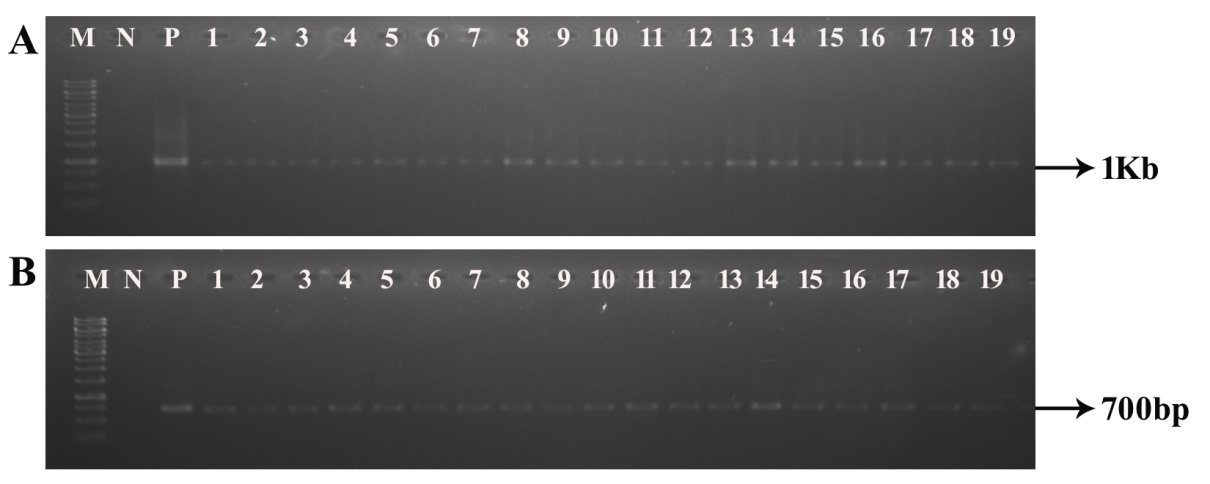


**Supplementary Figure 10:** PCR analysis of putative (T_1_) AtRd29A::OsSRDP transgenic PS2 plants using (**A**) *hptII* gene specific primers amplified a 1kb and (**B**) SRDP29A primer amplified a 700bp product. Amplicon present in 19 transgenic plants (Lane 1-19), Positive control (P-plasmid), amplicon absent in negative control (N- non transgenic-PS2), M-1kb ladder plus DNA marker.


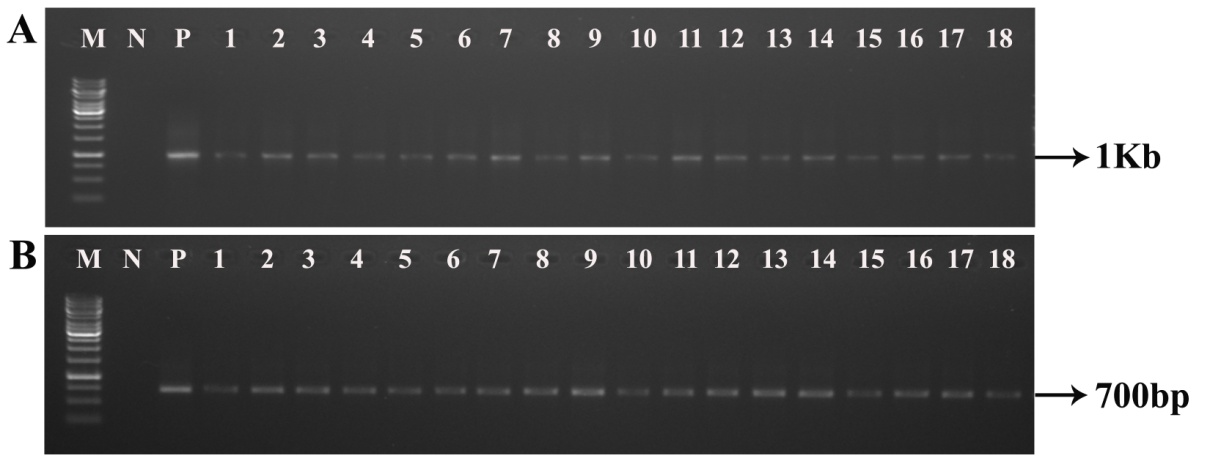


**Supplementary Figure11:** PCR confirmation of putative T_2_ AtRd29A::OsSRDP transgenic PS2 plants using (**A**) *hptII* specific primer amplified 1kb fragment and (**B**) SRDP29A primer amplified a 700bp product. Amplicon present in 18 transgenic plants (Lane 1-18), Positive control (P-plasmid), amplicon absent in negative control (N-Non transgenic-PS2), M-1kb ladder plus DNA marker.


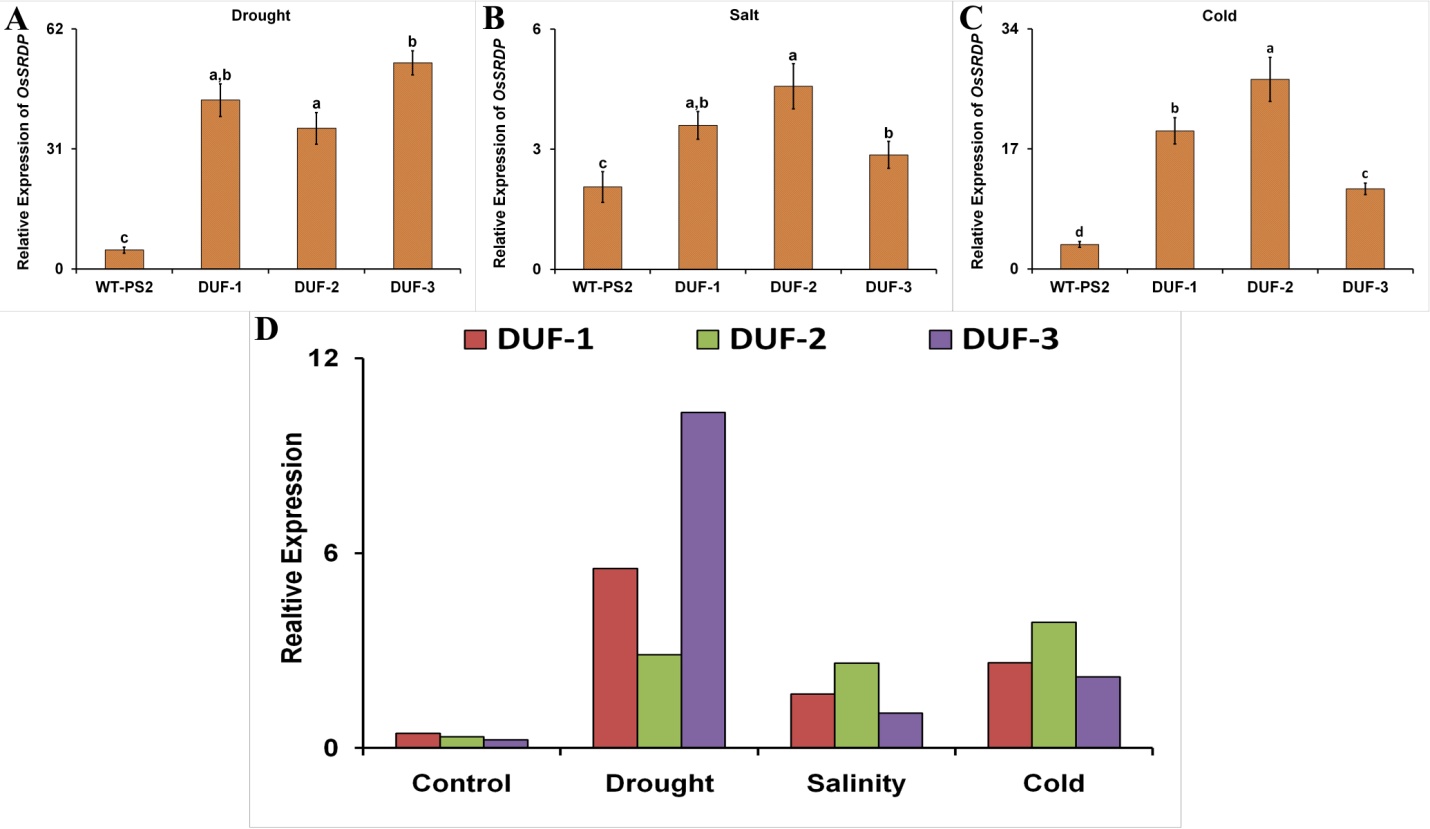


**Supplementary Figure 12: Analysis of transgene expression in AtRd29A::OsSRDP transgenic rice lines with WT plants under diverse abiotic stress.** (**A-C**) Relative expression analysis of *OsSRDP* in AtRd29A::OsSRDP transgenic rice lines with WT plants under drought, salt and cold stresses, respectively. (**D**) The expression of transgene in AtRd29A::OsSRDP transgenic lines under normal growth and diverse abiotic stress conditions using WT expression as the baseline. Each value represents mean of relative expression over three biological and three technical replicates, normalized with respect to *OsActin* was used as an internal control. Standard error of means (SD/√N; N=9) are used as error bars, and alphabets above the vertical bars represent statistically significant differences (Duncan’s Multiple Range Test: p ≤ 0.05) between WT (PS2) and AtRd29A::OsSRDP transgenic rice lines.


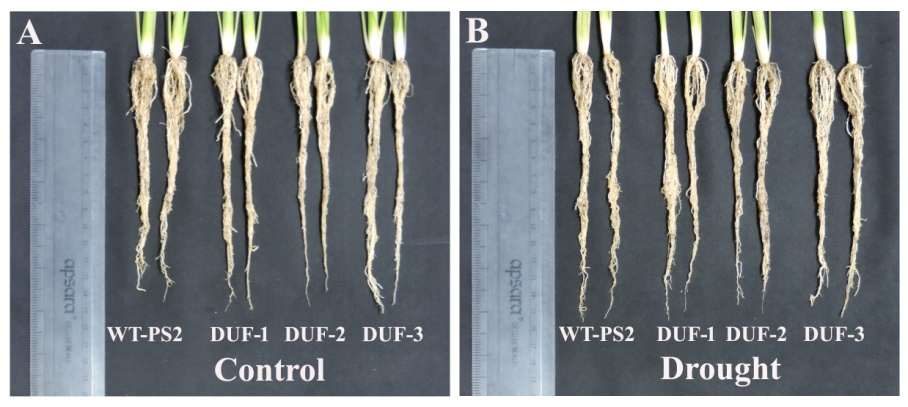


**Supplementary Figure13: Root morphology in response to drought treatment.** Comparison of root morphology from WT-PS2 and AtRd29A::OsSRDP transgenic lines under normal growth (**A**) and drought stress (**B**) conditions using 28d**-**old seedlings.

#
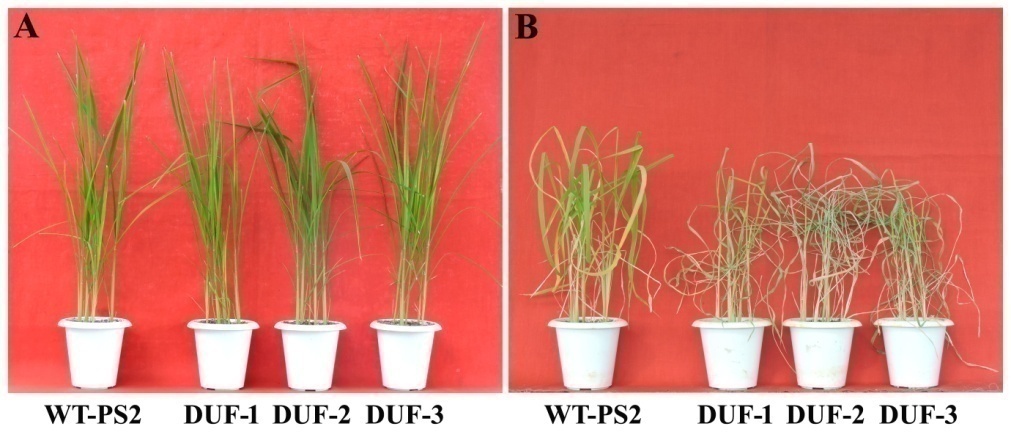


**Supplementary Figure14: Phenotypic changes in wild type and transgenic AtRd29A::OsSRDP rice plants under heat treatment.** (**A**) Phenotype of wild type and AtRd29A::OsSRDP transgenic rice seedlings grown under normal growth condition, (**B**) Four-five leaf stage of both rice seedlings imposed to heat treatment at 40°C for 2-3d.


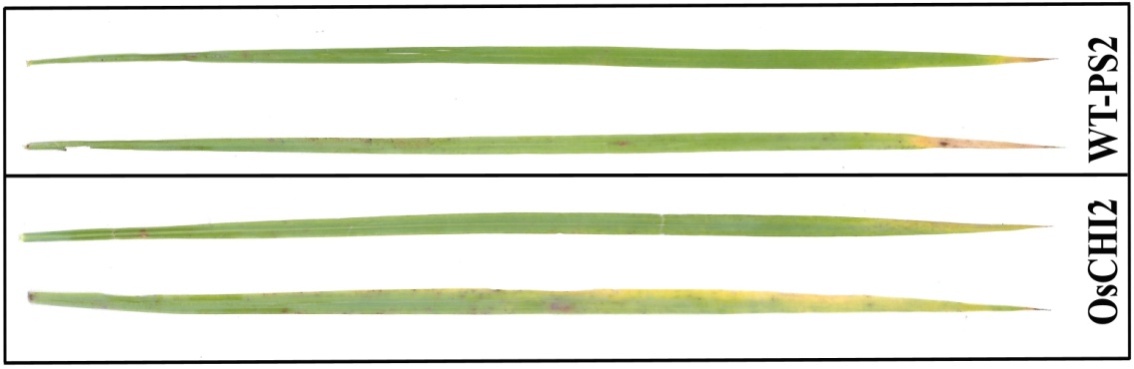


**Supplementary Figure 15: AtRd29A::OsCHI2 transgenic plants showed susceptible to rice blast disease.** Disease symptoms of WT-PS2 and AtRd29A::OsCHI2transgenic lines infected with *M. oryzae* on 21d old seedlings after 72hpi.


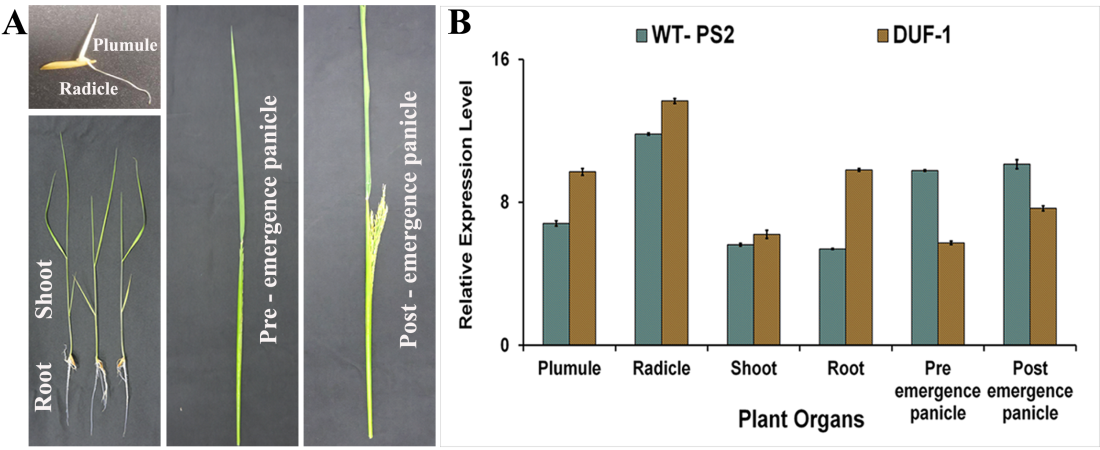


**Supplementary Figure 16: Different tissue/organs specific expression of *OsSRDP* gene.** (**A**) Various tissues including plumule, radicle, shoot, root, pre and post emergence panicle used for *OsSRDP* expression analysis, (**B**) Relative transcript abundance of the *OsSRDP* gene in different plant tissues. The organs examined were plumule, radicle, shoot, root, pre and post emergence panicle of wild type (PS2) and transgenic lines (AtRd29A::OsSRDP) grown under normal condition. The 2^-ΔCT^ method was used to determine the relative expression of *OsSRDP* with respect to that of the *OsActin* reference gene. The bars represent the mean values of three biological and three technical replicates and intervals over the bars indicate the standard error of the mean.

**
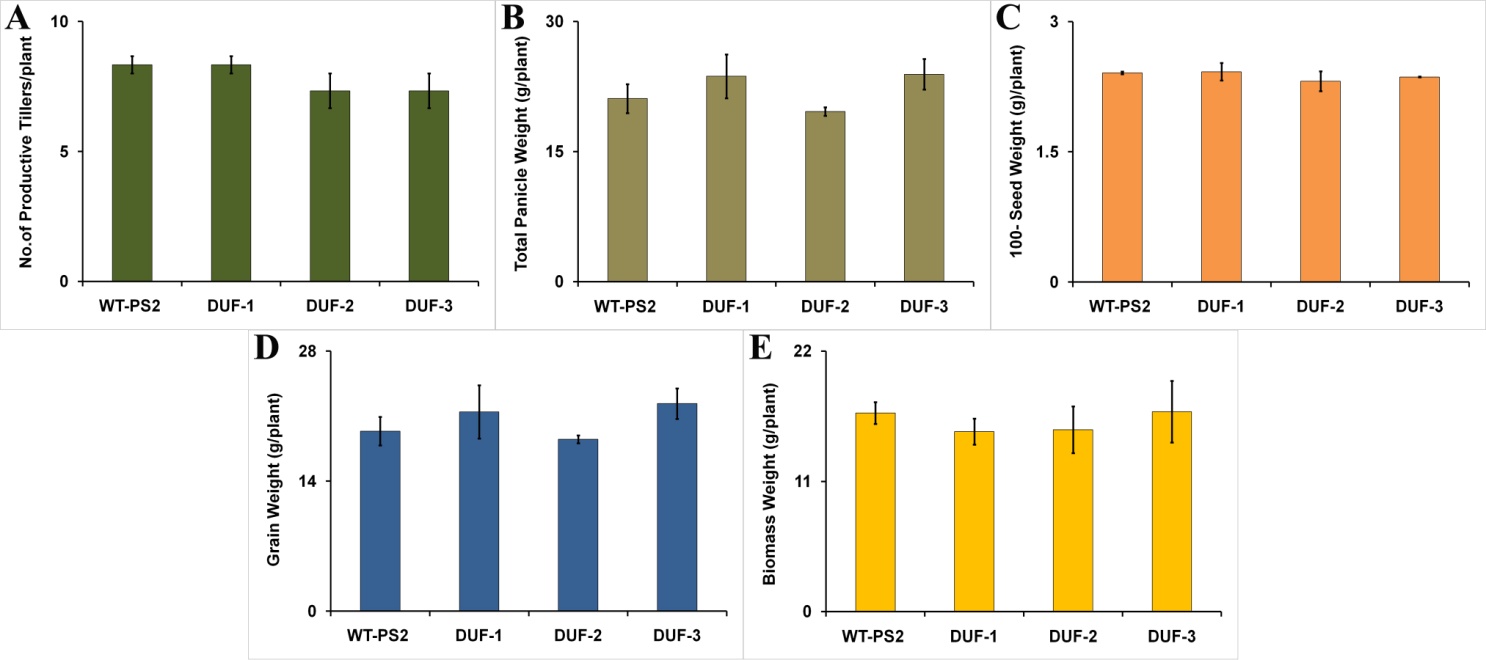
**

**Supplementary Figure 17: Comparative analysis of productivity related traits in wild type (PS2) and AtRd29A::OsSRDP transgenic rice plants under irrigated condition.** (**A**) Number of productive tillers, (**B**) Total panicle weight, (**C**) 100-seed weight, (**D**) Grain weight and (**E**) Biomass weight. The bars represent the mean values of fifteen independent replications and intervals above the bars indicate the standard error of the mean.
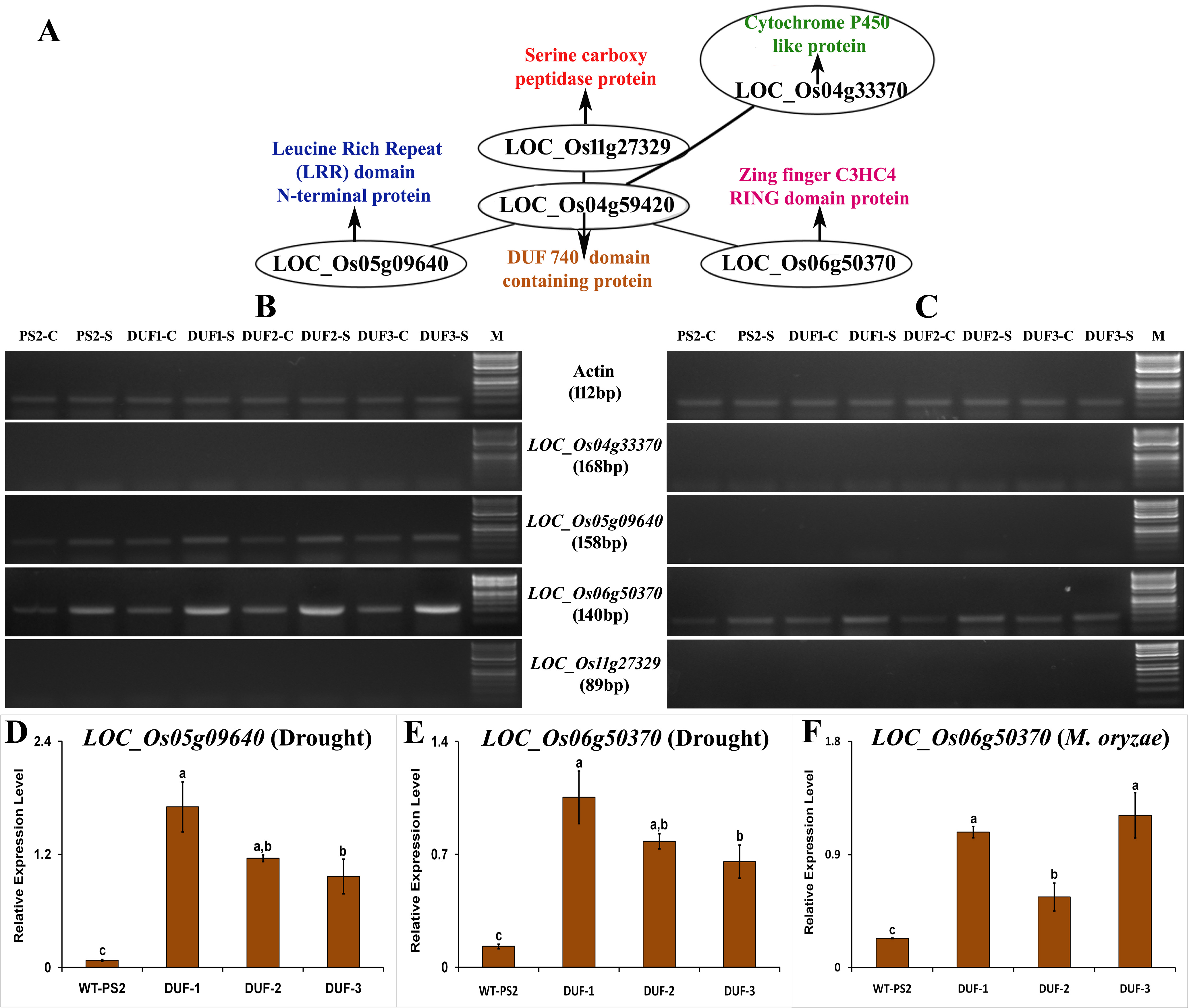


**Supplementary Figure18: Validation of co-expression gene(s) of *LOC_Os04g59420* in WT plants and AtRd29A::OsSRDP transgenic lines. (A)** Co-expression gene network of *LOC_Os04g59420* (*OsSRDP*) with its major and primary interacting partners. Semi-quantitative expression patterns of *LOC_Os04g33370, LOC_Os05g09640, LOC_Os06g50370* and *LOC_Os11g27329* genes in transgenic lines and WT plants under drought (**B**) and *M. oryzae* infection (**C**).There was no expression of LOC_Os04g33370 and LOC_Os011g27923 genes both in the transgenic lines and WT plants for either of the stresses namely, drought stress and *M*. *oryzae* infection. qRT-PCR analysis for expression profile of*LOC_Os05g09640* (**D)***,*and *LOC_Os06g50370* (**E**) under drought**,** and *LOC_Os06g50370*in response to *M. oryzae* infection (**F**). Each value represented as mean of relative expression over three biological and three technical replicates, normalized with respect to *OsActin* as an internal control. Standard error of means (SD/√N; N=9) are used as error bars, and alphabets above the vertical bars represent statistically significant differences (Duncan’s Multiple Range Test: p≤ 0.05) between transgenic rice lines (AtRd29A::OsSRDP) and WT (PS2).

**Supplementary Table 1:** Details of the primers sequence used in this study

| **Primer id** | **Forward primer sequence (5’-3’)** | **Reverse primer sequence (5’-3’)** |
| --- | --- | --- |
| **Sequencing assessment of the *OsSRDP* gene and its promoter** | | |
| OsSRDP_OL1 | GCAGGTATGGCACTGTACATATT | ACGTGGTCCAATTTCGATGG |
| OsSRDP_OL2 | TCTTCCCATCGTCCTTGCTT | TGTGCTGTTTAAAGGGTGTGT |
| OsSRDP_OL3 | CTGATCCGTTGACAAGCCAG | GCACTCAAGCATGGAGATCC |
| OsSRDP_OL4 | AGCTAGGTGATCAGTGAGTGT | TGCTAGGTCGGTATCTGGAG |
| OsSRDP_OL5 | GGTATGTCCTCTCCTCTTGGG | CGTGGTCGTCGTATCTGTCT |
| OsSRDP_OL6 | CCTTCAATTCTCTGTCGCTCG | CCTCGTCATTCCCAGTTGCA |
| OsSRDP_OL7 | TTCTTACCTCCCTCACTCTCTTC | CTTCAACCACTGGTCAAGCTAA |
| **Development of AtRd29A::OsSRDP transformants plants and molecular analysis** | | |
| *OsSRDP* | ATGAAGGATGGAGTAGCAGGAGGAGG | CTAGCTGAGAGGCATGGCGGGT |
| SRDP29A | AATACAATTCGAATGAGAAGG | AAGGAATCATCGAGGCTAG |
| Hpt-II | GGCGTCGGTTTCCACTATC | GCGACGTCTGTCGAGAAGTT |
| **For quantitative RT-PCR analysis** | | |
| *OsActin* | GATCTGGCATCACACCTTCTAC | CTGGGTCATCTTCTCACGATTG |
| *OsSRDP* | AAGACAGATACGACGACCAC | TTCTTGCCGTCGATCCC |
| *OsSOD* | TGATCTTGGAAAGGGTGG | TCGTAGAGTTTCAGGCTTC |
| *OsPOD* | AGCTAGATGACCATATACACG | GACACGACCATATACACTAC |
| *LOC_Os04g33370* | TACTCGTACCTCGACTCCCT | GATGGACGGGTTGTCCATGA |
| *LOC_Os05g09640* | CTGAGCTACAACTCGCTCTC | GAAGTTGAAGGAGACGGACA |
| *LOC_Os06g50370* | TTAATGTGATCTACAGCCTCCT | CTCGATCTCGAACAGGTCAG |
| *LOC_Os11g27329* | AAACCTGCAGAGGGAAATAC | GTTGATGTCCTTTGTGCAATC |

**Supplementary Table 2:** Segregation analysis of T_1_ progeny of rice *OsSRDP* transgenic lines

| **S. No** | **Transgenic Event** | **Total no**  **of T_1_ seeds** | **Hygromycin** | | **Segregation Ratio (χ^2^ value)** |
| --- | --- | --- | --- | --- | --- |
|  |  |  | **Resistant** | **Susceptible** |  |
| 1 | DUF-1 | 32 | 24 | 8 | 3:1 (0.000) |
| 2 | DUF-2 | 29 | 22 | 7 | 3:1 (0.0512) |
| 3 | DUF-3 | 32 | 24 | 8 | 3:1 (0.000) |
| 4 | DUF-4 | 31 | 29 | 2 | 15:1 (0.0176) |

**Supplementary Table 3:** Details of the DUF740 gene family members in rice

| **S. No** | **MSU ID** | **Clade** | **No of Domain(s)** | **Protein Length (AA)** | **Transposon Elements Insertion** | **Tissue Specific Expression** | **Expression under drought stress** | | |
| --- | --- | --- | --- | --- | --- | --- | --- | --- | --- |
|  |  |  |  |  |  |  | **Genotype** | **Growth stage** | **Fold change**  **(Log_2_)** |
| 1 | LOC_Os01g63310 | III-D2 | 1 | 651 | None | - | - | - | - |
| 2 | LOC_Os02g01080 | II-A | 2 | 354 | None | - | - | - | - |
| 3 | LOC_Os02g46420 | II-C2 | 1 | 290 | None | All Tissue | Bala | Vegetative | -1.45 |
| 4 | LOC_Os03g05460 | I-B | 2 | 445 | None | All Tissue | - | - | - |
| 5 | LOC_Os03g08970 | III-A | 1 | 176 | MITES | All Tissue | Dhaggadeshi | Seedling | 1.323 |
|  |  |  |  |  |  |  | Nipponbare | Seedling | 1.314 |
| 6 | LOC_Os04g49850 | II-C1 | 1 | 152 | None | Pre emergence inflorescence, pistil and embryo | - | - | - |
| 7 | LOC_Os04g59420 | I-A | 1 | 227 | None | - | IR20 | Seedling | 1.167 |
|  |  |  |  |  |  |  | Nagina22 | Reproductive | 1.195 |
| 8 | LOC_Os05g37800 | III-D1 | 1 | 662 | None | Pre, post, embryo, seed and pistil | - | - | - |
| 9 | LOC_Os06g11510 | III-B | 1 | 181 | Copia | Pre, post, embryo, seed, pistil and anther | - | - | - |
| 10 | LOC_Os10g41800 | II-B | 1 | 292 | None | - | - | - | - |
| 11 | LOC_Os11g48050 | III-C | 2 | 379 | None | - | - | - | - |

**Supplementary Table 4:** List of stress related cis-acting regulatory elements in the promoter region of the *OsSRDP* gene from N22 and PS2 genotypes

| **Name of motif** | **Sequence of motif** | **Function of the motif** | **Number of motif** | | | |
| --- | --- | --- | --- | --- | --- | --- |
|  |  |  | **N22** | | **PS2** | |
|  |  |  | **Sense** | **Reverse** | **Sense** | **Reverse** |
| CAT-box | GCCACT | Meristem expression related element | 1 | - | 1 | 1 |
| ABRE4 | CACGTA | Abscisic acid responsiveness element | 2 | 2 | 2 | 2 |
| G-box | TACGTG/  CACGTC | Light responsiveness element | 3 | 2 | 3 | 2 |
| MYB-RS (Recognition site) | CCGTTG | Water stress responsive element | 1 | - | 1 | - |
| CCGTCC-box | CCGTCC | Meristem specific activation element | 1 | 1 | 1 | 1 |
| AT~TATA-box | TATATA | Light responsive element | 2 | 2 | 2 | 2 |
| ABRE | ACGTG | Abscisic acid responsiveness element | 2 | 3 | 2 | 3 |
| as-1 (activation sequence-1) | TGACG | Different abiotic stress responsive element | 1 | - | 1 | - |
| Box 4 | ATTAAT | Part of a conserved DNA module involved in light responsiveness | 4 | 2 | 4 | 2 |
| MeJARE | TGACG | Methyl jasmonate responsiveness element | 1 | - | 1 | - |
| MYB-LS (Like Sequence) | TAACCA | - | 1 | 1 | 1 | 1 |
| ABRE3a | TACGTG | Abscisic acid responsiveness element | 2 | 2 | 2 | 2 |
| P-box | CCTTTTG | Gibberellin responsive element | 1 | - | 1 | - |
| GT1-motif | GGTTAA | Light responsive element | 1 | - | 1 | - |
| CCGTCC-motif | CCGTCC | Meristem specific activation element | 1 | 1 | 1 | 1 |
| A-box | CCGTCC | Cis-acting regulatory element | 1 | 1 | 1 | 1 |
| MYB | CAACCA/  TAACCA | Drought responsiveness element | 3 | 2 | 3 | 2 |
| Myb | TAACTG/CAACTG | Drought responsiveness element | 1 | 1 | 1 | 1 |
| MYC | CATTTG | Drought responsiveness element | 2 | 2 | 2 | 2 |
| DRE core | GCCGAC | Cold and dehydration response element | 1 | - | 1 | - |
| W box | TTGACC | Salicylic acid responsiveness element | - | 1 | - | 1 |
| TCA-element | CCATCTTTTT | Salicylic acid responsiveness element | - | 2 | - | 1 |
| CGTCA-motif | CGTCA | Methyl jasmonate responsiveness element | - | 1 | - | 1 |
| WRE3 | CCACCT | Wounding and pathogen response element | - | 1 | - | 1 |
| box S | AGCCACC | - | - | 1 | - | 1 |
| RY-element | CATGCATG | Seed-specific regulation element | - | 2 | - | 2 |
| CCAAT-box | CAACGG | MYBHv1 binding site | - | 1 | - | 1 |
| MBS | CAACTG | MYB binding site involved in drought-inducibility | - | 1 | - | 1 |
| STRE | AGGGG | Multiple stresses responsive element | - | 1 | - | 1 |
| ARE | AAACCA | Anaerobic induction essential element | - | 2 | - | 2 |
| GATA-motif | AAGGATAAGG | Light responsive element | - | 1 | - | 1 |
| AAGAA-motif | GAAAGAA | Secondary xylem development response element | - | 1 | - | 1 |
| AP-1 (Activator Protein) | TGAGTTAG | Stress responsive element | - | 1 | - | 1 |

**Supplementary Table 5:** Comparative analysis of prediction structure of the OsSRDP protein in N22 and PS2 genotypes

| **Enzyme commission (EC) numbers and active sites** | | | | | | | | | | | | | | | | | | |
| --- | --- | --- | --- | --- | --- | --- | --- | --- | --- | --- | --- | --- | --- | --- | --- | --- | --- | --- |
| **Rank** | **Nagina22** | | | | | | | | | **Pusa Sugandh 2** | | | | | | | | |
|  | **EC Number (Name)** | | | | | | **C-score ^EC^** | | | **EC Number (Enzyme Name)** | | | | | | **C-Score ^EC^** | | |
| 1 | 4.4.1.16 (Selenocysteinelyase) | | | | | | 0.114 | | | 2.8.1.7 (Cysteine desulfurase) | | | | | | 0.077 | | |
| 2 | 2.8.1.7 (Cysteine desulfurase) | | | | | | 0.113 | | | 2.6.1.44 (Alanine glyoxylate transaminase) | | | | | | 0.076 | | |
| 3 | 4.4.1 (Carbon-sulfur lyases) | | | | | | 0.109 | | | 4.4.1.16 (Selenocysteinelyase) | | | | | | 0.075 | | |
| 4 | 3.7.1.3 (Kynureninase) | | | | | | 0.105 | | | 3.7.1.3 (Kynureninase) | | | | | | 0.075 | | |
| 5 | 2.6.1 (Transaminases) | | | | | | 0.105 | | | 3.7.1.3 (Kynureninase) | | | | | | 0.073 | | |
| **Analysis of ligand binding** | | | | | | | | | | | | | | | | | | |
|  | | | **Nagina 22** | | | | | | | **Pusa Sugandh 2** | | | | | | | | |
| **Name of Ligand** | | | Pyridoxal 5'-phosphate | | | | | Ligand-Zn | | Pyridoxal 5'-phosphate | | | | Ligand-Zn | | | | |
| **Cluster size** | | | 33 | | | | | 9 | | 20 | | | | - | | | | |
| **C-Score** | | | 0.36 | | | | | 0.10 | | 0.32 | | | | - | | | | |
| **Binding sites**  **residuces** | | | 20,21,22,25,71,117,  119,146,148,149, 171,172 | | | | | 19,22,147 | | 20,21,22,70,115,118,145,  147,148, 170,171 | | | | - | | | | |
| **Prediction of gene ontology** | | | | | | | | | | | | | | | | | | |
| **Nagina22** | | | | | | | | | | **Pusa Sugandh 2** | | | | | | | | |
| **Molecular**  **Function** | | **GO Score** | | **Biological Process** | **GO Score** | **Cellular**  **Component** | | | **GO**  **Score** | | **Molecular**  **Function** | **GO Score** | **Biological Process** | | **GO Score** | | **Cellular**  **Component** | **GO**  **Score** |
| Pyridoxal phosphate binding (GO:0030170) | | 0.44 | | Sulfur amino acid metabolic  process  (GO:0000096) | 0.43 | Obsolete intracellular part (GO:0044424) | | | 0.58 | | Pyridoxal phosphate binding  (GO:0030170) | 0.32 | Metabolic process  (GO:0008152) | | 0.32 | | Cytoplasm  (GO:0005737) | 0.14 |
| Sulfur-transferase activity (GO:0016783) | | 0.43 | |  |  |  |  |  |  |  | Catalytic activity  (GO:0003824) | 0.32 |  |  |  |  |  |  |
|  |  |  |  | Serine family amino acid metabolic  process  (GO:0009069) | 0.43 |  |  |  |  |  |  |  |  |  |  |  |  |  |
| Carbon-sulfur lyase activity  (GO:0016846) | | 0.42 | |  |  |  |  |  |  |  |  |  |  |  |  |  |  |  |
